# Crossovers are regulated by a conserved and disordered synaptonemal complex domain

**DOI:** 10.1093/nar/gkaf095

**Published:** 2025-02-18

**Authors:** Ana Rita Rodrigues Neves, Ivana Čavka, Tobias Rausch, Simone Köhler

**Affiliations:** Cell Biology and Biophysics Unit, European Molecular Biology Laboratory (EMBL), 69117 Heidelberg, Germany; Collaboration for joint PhD degree between EMBL and Heidelberg University, Faculty of Biosciences, 69117 Heidelberg University, Heidelberg, Germany; Cell Biology and Biophysics Unit, European Molecular Biology Laboratory (EMBL), 69117 Heidelberg, Germany; Collaboration for joint PhD degree between EMBL and Heidelberg University, Faculty of Biosciences, 69117 Heidelberg University, Heidelberg, Germany; Genome Biology Unit, European Molecular Biology Laboratory (EMBL), 69117 Heidelberg, Germany; GeneCore, European Molecular Biology Laboratory (EMBL), 69117 Heidelberg, Germany; Cell Biology and Biophysics Unit, European Molecular Biology Laboratory (EMBL), 69117 Heidelberg, Germany

## Abstract

During meiosis, the number and distribution of crossovers (COs) must be precisely regulated through CO assurance and interference to prevent chromosome missegregation and genomic instability in the progeny. Here we show that this regulation of COs depends on a disordered and conserved domain within the synaptonemal complex (SC). This domain is located at the C-terminus of the central element protein SYP-4 in *Caenorhabditis elegans*. While not necessary for synapsis, the C-terminus of SYP-4 is crucial for both CO assurance and interference. Although the SYP-4 C-terminus contains many potential phosphorylation sites, we found that phosphorylation is not the primary regulator of CO events. Instead, we discovered that nine conserved phenylalanines are required to recruit a pro-CO factor predicted to be an E3 ligase and regulate the physical properties of the SC. We propose that this conserved and disordered domain plays a crucial role in maintaining the SC in a state that allows transmitting signals to regulate CO formation. While the underlying mechanisms remain to be fully understood, our findings align with existing models suggesting that the SC plays a critical role in determining the number and distribution of COs along chromosomes, thereby safeguarding the genome for future generations.

## Graphical Abstract

**Graphical Abstract:**
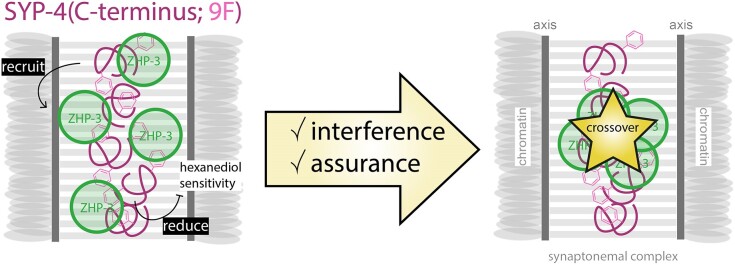

## Introduction

Meiosis is crucial for sexual reproduction since it segregates not only sister chromatids but also homologous chromosomes to generate gametes with half the original ploidy. Errors in the segregation of homologous chromosomes are a major cause of aneuploidy, which can lead to infertility, congenital conditions, and miscarriages [1, 2].

The successful separation of homologous chromosomes during meiosis I depends on the formation of crossovers (COs) between the homologs. COs not only increase the genetic diversity of the gametes but also create a physical link between the chromosomes known as chiasma. These chiasmata hold the homologs together until anaphase I and in their absence homologous chromosome segregation is impaired [3–5]. The formation of COs is initiated by the introduction of DNA double-strand breaks (DSBs) that are repaired by homologous recombination. However, only a small subset of DSBs is processed into actual COs as an excessive number of COs can be deleterious [6, 7]. Three primary mechanisms regulate CO formation in meiosis: CO assurance ensures that each pair of homologs receives at least one obligate CO; CO interference establishes a nonrandom distribution of COs, with neighbouring COs spaced further apart than expected by chance; and CO homeostasis maintains a constant number of COs within each meiotic nucleus to safeguard the system against deficiencies or surpluses of DSBs [8]. These highly regulated—or class I—COs require ZMM proteins which are named for their members in budding yeast, Zip1/2/3/4, Msh4/5, and Mer3. Additionally, many organisms may also acquire a small number of noninterfering class II COs that are formed independently of the class I CO pathway and may function as an alternative repair pathway for surplus DSBs [9–11]. While the regulation of essential class I COs through assurance, interference, and homeostasis is widespread across the tree of life, the precise mechanisms by which CO formation is controlled remain unclear.

Specifically, the role of the synaptonemal complex (SC) in regulating CO formation is a topic of ongoing debate. Through the process of synapsis, this protein structure brings the paired homologous chromosomes into close proximity of around 100 nm [12, 13]. The SC is formed by several interacting coiled-coil (CC) proteins. In the nematode *Caenorhabditis elegans*, the SC consists of eight interdependent proteins: six CC proteins, SYP-1-6, and two Skp1-related proteins, SKR-1/2 [14–20]. While the SC is crucial for CO formation in most species, its exact role in the precise patterning of class I COs is still ambiguous. In budding yeast, CO patterning is established independently of SC formation, though SC formation is necessary for the completion of CO formation [21, 22]. In contrast, COs are formed in the absence of SC in *A. thaliana*, but CO patterning strictly depends on the SC with a complete loss of CO assurance and interference in absence of the SC [23, 24]. Similarly, earlier studies in *C. elegans*, where only class I COs are observed under normal circumstances [10], demonstrated that defects in SC assembly by partial depletion or mutation of its components also give rise to reduced interference [18, 25, 26].

The interdependence of SC integrity and CO patterning in *A. thaliana* and *C. elegans*, along with the liquid-like behaviour of the SC, has led to the proposal that an SC-dependent ’coarsening’ mechanism establishes CO assurance and interference [23, 24, 27–31]. In this model, a pro-CO factor, presumably the essential ZMM protein and Zip3 homolog ZHP-3 in *C. elegans*, or HEI10 in *A. thaliana*, initially loads along the SC using its liquid-like properties to diffuse along synapsed chromosomes [27, 28], before accumulating at designated CO sites [32, 33]. Indeed, recent single molecule tracking experiments directly confirmed the diffusion of ZHP-3 along SCs [34]. At the same time, ZHP-3 can bind to recombination intermediates where it directly interacts with the *C. elegans* CO marker protein COSA-1, a cyclin-like protein necessary for CO formation [35–37]. Eventually, ZHP-3/HEI10 undergoes a coarsening process that selects a few widely spaced recombination intermediates to become designated CO sites [29, 30].

In *A. thaliana*, the coarsening model predicts the loss of both assurance and interference when the coarsening factor HEI10 is not confined to SCs along paired homologous chromosomes in synapsis-deficient mutants. In such cases, HEI10 is thought to diffuse and coarsen *in trans* across all chromosomes in the nucleus in a dosage-dependent manner, rather than *in cis* along individual chromosomes, resulting in a random distribution of CO events [23, 24, 30, 31, 38]. However, the model fails to explain why interference is detected before SC formation in budding yeast [21, 22]. Therefore, it remains unclear whether the observed coarsening process is the actual cause or just a consequence of CO designation, or whether different species use different regulatory mechanisms to limit CO designation.

Furthermore, directly testing the effect of the SC on CO regulation remains challenging, since CO formation depends on synapsis in *C. elegans* and most other model organisms [17, 39–41]. Moreover, the molecular mechanism by which the potential coarsening factor is recruited to the SC remains unknown.

In this study, we show that the last 114 amino acids of SYP-4 are crucial for regulating CO formation during meiosis in *C. elegans*. Deleting this region, or mutating conserved phenylalanines within, allows for synapsis but disrupts both CO assurance and interference, resulting in increased embryonic lethality. We identify a link between these CO defects and the failure to localize ZHP-3 to the SC, accompanied by changes in the biophysical properties of the SC. Our findings indicate that the C-terminus of SYP-4 modulates the biophysical characteristics of the SC and is required to recruit ZHP-3 to the SC, and we propose that one or both of these properties may contribute to the essential role of the SC in CO regulation. We propose the C-terminus of SYP-4 as a critical regulator of CO formation, and this function is likely conserved across species.

## Materials and methods

### Maintenance and culture conditions of *C. elegans* strains

All *C. elegans* strains were cultured at 20°C under standard conditions on nematode growth medium plates inoculated with *Escherichia coli* OP50 [42]. All experiments were performed using young adults 18–24 h post-L4. For experiments with balanced strains, we selected homozygous nongreen fluorescent or heterozygous green fluorescent L4-staged animals as indicated. A list of all strains used in this study can be found in Supplementary Table S1.

### Generation of *C. elegans* strains by CRISPR/Cas9-mediated genome editing

New alleles in this study were generated by CRISPR/Cas9-mediated genome editing by injecting preassembled Cas9-gRNA (guide RNA) ribonucleoprotein (RNP) complexes as described previously [43]. The Cas9-NLS protein was purchased either from the EMBL Protein Expression and Purification Core Facility or from IDT. The common tracrRNA (trans-activating CRISPR RNA) as well as all crRNAs (CRISPR RNA) listed in Supplementary Table S2 were purchased from IDT. Wild-type sequences were obtained from WormBase [44] and modified to yield the repair template sequences used to generate each new allele as listed in Supplementary Table S3. For the *ske19* and *ske71* alleles, short single-stranded DNA oligos (Sigma–Aldrich) were used as repair templates. For the *ske25* allele, a biotinylated-double-stranded DNA (dsDNA) obtained by polymerase chain reaction (PCR) from a gBlock (IDT) was used as a repair template. For the *ske61* allele, a dsDNA was obtained by PCR from pCFJ1415 [45]. For all remaining alleles the repair templates were obtained by PCR from gBlocks (IDT). To enrich for successfully injected progeny, two plasmids carrying red fluorescent transgenes {2.5 ng/μl pCFJ90 (Pmyo-2::mCherry) and 5 ng/μl pCFJ104 (Pmyo-3::mCherry) [46]} were co-injected with the Cas9-gRNA RNP complex and repair template. Individual F1s expressing either of the two plasmids were picked onto new plates and were screened for correctly edited genes by PCR two or more days later (Supplementary Table S4). For alleles containing point mutations in *syp-4*, we also introduced or removed restriction sites to distinguish mutant from wild-type alleles (Supplementary Table S4). All sequences were confirmed by Sanger sequencing (Eurofins Genomics). All epitope-tagged *syp-4*, *zhp-3*, *rad-51*, and *cosa-1* wild-type genes generated in this study were functional with no major increases in embryonic lethality or male progeny (Supplementary Table S5). Strains containing new alleles were outcrossed at least twice, or experiments were performed using at least two synonymous lines derived from different CRISPR/Cas9-editing events. To generate a balancer for *syp-4*, the essential gene *let-383* was replaced by a codon optimized green fluorescent protein (GFP) transgene in the Hawaiian CB4856 background. This allele was then introgressed into the Bristol N2 background by nine consecutive crosses.

### Viability assays

To determine the viability of eggs, L4 hermaphrodites were singled onto individual plates to lay eggs. After each laying period of 12–24 h, the number of fertilized eggs was counted, and the individual worms were transferred to new plates. This process was repeated until they stopped laying eggs. The surviving progeny was counted once they reached the adult stage.

### SYP-4 multiple sequence alignment, amino acid conservation and disorder prediction

We searched for homologs of the *C. elegans* SYP-4 protein sequence (uniprot ID Q9N5K3) in all proteomes listed in Supplementary Table S6 using a protein hidden Markov model (phmmer). The hits obtained from this first search were used to perform a reciprocal mapping by searching them against the *C. elegans* proteome. Only hits that successfully mapped back to *C. elegans* SYP-4 were then used to perform a multiple sequence alignment (MSA) using MAFFT (Multiple Alignment using Fast Fourier Transform) with the genafpair algorithm and a maximum of 1000 iterations [47]. Both amino acid conservation scores and protein disorder prediction were retrieved from JalView [48]. The conservation scores correspond to the calculated quality scores based on BLOSUM62 substitution matrix. Protein disorder scores are based on IUPred prediction for short regions. PairCoil2 [49] was used to predict CC motifs in SYP-4 protein sequence (P-score > 0.025).

### Immunofluorescence

Animals were dissected 24 h post-L4 and stained as described previously in [50] with modifications described in [51] and mounted in ProLong Glass antifade mounting medium (Invitrogen, P36984).

The following primary antibodies were used: chicken anti-HTP-3 (1:500 [52]), rabbit anti-SYP-5 (1:500 [18]), mouse anti-HA (1:1250, Invitrogen, $\#$A190-138A), or mouse anti-V5 (1:500, Invitrogen, $\#$R960-25). As secondary antibodies the following were used: Alexa Fluor 488 donkey anti-chicken (1:500, Jackson ImmunoResearch, 703-545-155), Alexa Fluor 546 goat anti-chicken (1:500, Invitrogen, $\#$A-11040), Alexa Fluor 546 donkey anti-rabbit (1:500, Invitrogen, $\#$A10040), Alexa Fluor 546 donkey anti-mouse (1:500, Invitrogen, $\#$A10036), Alexa Fluor 647 donkey anti-mouse (1:500, Jackson ImmunoResearch, 715-605-150). Sample blocking and antibody incubation were performed in 1× Roche blocking solution in PBST (phosphate-buffered saline with 0.1 % Tween 20). To visualize COSA-1 foci, animals carrying a *HaloTag::cosa-1* allele from well-fed plates were incubated overnight with 6.67 μM JF669 HaloTag ligand [53] (Lavis Lab, JBG-27-075A) or 1.3 μM JF646 HaloTag ligand (Promega, GA1120) in a 20 μl droplet of M9 buffer containing *E. coli* OP-50 and 0.2% (v/v) Tween-20. To assess the loading of SYP-4 or SYP-5 throughout meiosis and test the sensitivity of SYP-4 to 4% (v/v) 1,6-hexanediol, both *syp-4* mutant animals carrying the endogenously tagged *HaloTag::cosa-1* allele and control animals with untagged *cosa-1* were fed overnight on plates seeded with OP50 *E. coli* in a 20 μl drop of 6.67 μM JF669 HaloTag ligand (Lavis Lab, JBG-27-075A) in M9 buffer with 0.2% (v/v) Tween-20 and dissected together on the same slide the next day. For hexanediol treatments, the dissected samples were incubated for 30 s with 4% (v/v) 1,6-hexanediol before fixation as described in [27]. Images were acquired within two weeks after mounting. At least two independent immunofluorescence experiments were performed.

### Fluorescence microscopy

For quantification of COSA-1 foci and 4′,6-diamidino-2-phenylindole (DAPI) staining bodies, 0.16 μm-spaced *z*-stacks were acquired on a Zeiss LSM 880 Airyscan laser scanning confocal microscope with a 63× 1.4 NA DIC M27 oil plan-apochromat objective using the Airyscan FAST mode. The images were deconvolved using the 3D auto mode from the Airyscan processing tool available in the Zen Black v14.0.15.201 software (Zeiss).

For all other experiments, 0.16–0.20 μm-spaced *z*-stacks were acquired on an Olympus spinning disk confocal system IXplore SpinSR with a 60× 1.42 NA oil plan-apochromat objective using the cellSens software (Olympus). For quantification of SYP-4, SYP-5, or ZHP-3 loading throughout pachytene and hexanediol experiments images were acquired using the higher resolution SoRa disk with a 3.2× magnification. For ‘synapsis zone’ quantification, images were acquired using a 50 μm disk with a 1× magnification. Both disks were used for quantification of RAD-51 foci.

All image manipulations, such as maximum intensity projection, tile stitching, channel intensity adjustment, and colour correction were performed using the free software Fiji [54].

### Image quantification

#### Segmentation of meiotic nuclei

DAPI-stained meiotic nuclei were blurred with a 2.5 blurring factor and segmented in 3D using a custom pipeline based on the Cellpose2.0 neural network [55] using a custom model (https://github.com/KoehlerLab/Cellpose_germlineNuclei/blob/main/Cellpose_germlineNuclei/cellpose_germlineNuclei_KoehlerLab) as described in [56]. To remove incomplete nuclei, segmented nuclei masks touching the first or last slice in *z* were disregarded.

#### Quantification of SYP-4/5 and ZHP-3 loading throughout meiosis and SYP-4 sensitivity to 1,6-hexanediol

We used custom Python and R scripts to quantify the loading of SYP-4, SYP-5, and ZHP-3 along the axis, or the sensitivity of SYP-4 to 1,6-hexanediol (https://github.com/KoehlerLab/SYPquant/tree/main/SYPquant/SCintensityAnalysis). To calculate the background signal of SYP-4::HA, SYP-5, or ZHP-3::V5 labelling, we calculated the average pixel intensity within 10 and 50 pixels from the segmented objects (including filtered out objects). To quantify the SYP-4::HA, SYP-5, or ZHP-3::V5 signal along the chromosome axes, we segmented the HTP-3 signal within each segmented nucleus using the mean thresholding method from the Python library scikit-image v0.19.1 [57]. For each nucleus, the total intensities of SYP-4::HA, SYP-5, or ZHP-3::V5 on the HTP-3 mask and in the nucleus were calculated after subtracting the average background signal. For the quantification of SYP-4::HA or SYP-5 loading on the axes, the total intensity of SYP-4::HA, or SYP-5, on the axes was normalized to the average total intensity of SYP-4::HA/SYP-5 signal on the axes in wild-type samples from the same slide. For the quantification of ZHP-3::V5 loading or SYP-4::HA sensitivity to 1,6-hexanediol, we calculated the ratio between the intensity on the axes and the total intensity in the nucleus.

A region of interest was manually defined for each germline in *xy* from the beginning of transition zone to the end of pachytene, and objects outside this region were removed. To remove potential fragmented meiotic nuclei, apoptotic nuclei or somatic nuclei, only segmented nuclei with a volume between 10 and 60 μm^3^ (excluding), a sphericity higher than 0.4 and an intensity ratio between 0.3 and 0.8 (including) were considered. To assess the loading and sensitivity of SYP-4, SYP-5, or ZHP-3 along the pachytene region, the germlines were straightened by applying a Loess function to the centroid positions of all segmented nuclei, and oriented from left (early) to right (late). The germline length was normalized from the beginning of the transition zone (SYP-4::HA and SYP-5 loading) or beginning of pachytene (hexanediol sensitivity and ZHP-3::V5 loading) to the end of pachytene using manual annotations.

#### Quantification of RAD-51

For the quantification of V5::RAD-51 foci, we ran SpotMAX v0.9.4, a 3D gaussian fitter software suitable for the identification of 3D foci [58], on segmented nuclei along the entire germline. The foci were initially thresholded inside the segmentation masks using the Yen thresholding method and fit by a 3D gaussian function. A region of interest was manually defined in *xy* for each germline from the beginning of the proliferative zone to the end of pachytene and objects outside this region were removed. To quantify the average number of V5::RAD-51 per nucleus along the germline, the germlines were straightened by applying a Loess function to the centroid positions of all segmented nuclei, and oriented from left (early) to right (late). The germline length was normalized from the beginning of the proliferative zone to the end of pachytene using manual annotations, and only nuclei with a diameter between 1 and 6 μm were considered.

#### Quantification of COSA-1

For the quantification of COSA-1 foci, well segmented nuclei in late pachytene were manually selected. We then used SpotMAX v0.8.0 [58] to quantify the number of foci per segmented meiotic nucleus. The foci were initially thresholded inside the segmentation masks using the Otsu thresholding method and fit by a 3D gaussian function.

#### Chromosome tracing

The segmented nuclei were first cropped using the object’s bounding box. Each of the six chromosome filaments were semi-manually traced using the Fiji plugin BigTrace (v.0.05 or v.0.2.1) on semi-automatic mode (https://github.com/ekatrukha/BigTrace) based on the SYP-4::HA signal. For visualization purposes, traced chromosomes were straightened using an 11-pixel wide line around the centre line using BigTrace (v.0.2.1) and the respective maximum intensity projection of the merged SYP-4 and COSA-1 channels was generated.

#### COSA-1 localization along the chromosomes

The COSA-1 centroid position was mapped to the position along the traced chromosome using a custom Python script (https://github.com/KoehlerLab/SYPquant/tree/main/SYPquant/ChrTraceAnalysis) that comprises the following steps: (i) calculation of the global offset between the COSA-1 and SYP-4 channel to correct the COSA-1 centroid coordinates; (ii) spline fitting of BigTrace’s output (trace point coordinates) and sampling of equidistant points along the interpolated spline; (iii) mapping of the COSA-1 centroid positions onto the closest trace corresponding to the point on the trace with the smallest Euclidean distance to the COSA-1 centroid position. We acknowledge that while this mapping procedure generally performs well, it is possible that some foci may be mis-mapped due to chromatic aberrations between the two channels.

#### Quantification of DAPI-staining bodies

The number of DAPI-staining bodies in diakinesis was quantified manually in 3D stacks from DAPI-counterstained samples using Fiji. Counts include DAPI-staining bodies of all shapes (univalents, ‘normal’ bivalents, and ring-shaped bivalents).

#### Quantification of the duration of synapsis

The duration of synapsis was quantified manually in maximum intensity projections using Fiji. The length of the ‘synapsis zone’ was defined from the first nuclei with visible HTP-3 stretches until the last row of nuclei with incomplete synapsis based on HTP-3 and SYP-4::HA immunofluorescence signal localization.

### Recombination mapping

#### Sample preparation

To map CO frequencies genetically, L4-staged hermaphrodites of different genotypes in the Bristol background [wild-type N2 or *syp-4(9FA)::ha (ske39-1)/skeIR1* I; *halo::cosa-1 (ske25)* III] were crossed to males of the same genotype in the Hawaiian background [wild-type CB4856 and *syp-4(9FA)::ha (ske39-2)/ske61* I, respectively]. The F1 hermaphrodite Bristol/Hawaiian progeny [N2/CB4856 or *syp-4(9FA)::ha (ske39-1)*/*syp-4(9FA)::ha (ske39-2)* I; *halo::cosa-1 (ske25)*/+ III] was crossed with male homozygous CB4856 Hawaiian animals, and 24 h later hermaphrodite animals were transferred to empty 6-cm nematode growth medium agar plates containing a 20 μl drop of 10 mM serotonin [Sigma–Aldrich, H7752] within a palmitic acid ring to increase the rate of egg laying and prevent worms from leaving the agar plate, respectively [59, 60]. Single F2 embryos were collected for a period of 6 h followed by DNA isolation, library preparation and whole genome sequencing. Singled F2 embryos were transferred onto lysis buffer [1× DreamTaq Buffer (Thermo Scientific, B65), 1 mg/ml Proteinase K (EMBL Protein Expression and Purification Core Facility)] and stored at −80°C for at least 24 h. The genomic DNA was isolated after a 1-h incubation at 65°C followed by Proteinase K inactivation at 95°C for 15 min. The genomic DNA was amplified using the PicoPLEX^®^ Single Cell WGA Kit (TAKARA, R300672) according to the manufacturer’s instructions. The amplified genomic DNA was purified using a 1.8× volume of SPRIselect beads (Beckman Coulter, B23319) followed by Tn5-based tagmentation and 300PE Illumina sequencing on an Illumina NextSeq 2000 platform (EMBL Gene Core Facility) to a final coverage of 1.8-6.7× per sample.

#### Data processing

The sequencing quality was assessed using FastQC (https://www.bioinformatics.babraham.ac.uk/projects/fastqc/) and MultiQC [61]. The adapter sequences from Illumina index primers were removed using Trimmomatic [62]. Reads mapping to the standard-8 database (10/9/2023) available at https://benlangmead.github.io/aws-indexes/k2 were filtered out using Kraken2 [63]. The remaining reads were mapped to the softmasked N2 Bristol (BioProject PRJNA13758) and CB4856 Hawaiian (BioProject PRJNA275000) WS288 genome releases available on WormBase (https://wormbase.org) [44] using BWA-MEM [64]. Read sorting, duplicate removal and read filtering were performed using SAMtools [65]. Reads with a mapping quality score below ten and mitochondrial reads were not considered.

#### Data analysis

To identify CO sites genetically, we counted the number of reads in 5-kb windows that mapped exclusively to the N2 Bristol or CB4856 Hawaiian genome, respectively, using SAMtools [65] and Alfred [66]. To infer genotypes, we calculated the gliding average of the ratio between Bristol-specific reads and the total number of Bristol- and Hawaiian-specific reads across 100 5-kb windows. If this ratio was >0.9, we classified it as homozygous Bristol (only observed for haploid chromosomes), and if it was <0.25, we classified it as homozygous Hawaiian. Ratios in between indicated a heterozygous genotype. We identified COs as points where there was a change in genotype. We only considered transitions supported by >1500 reads, with consistent genotypes on either side for at least 15 kb. Each CO site was manually verified.

Recombination sites on chromosome I were excluded from further analysis due to the specific recombination events observed in *syp-4(9FA)::ha (ske39-1)*/*syp-4(9FA)::ha (ske39-2)* I; *halo::cosa-1 (ske25)*/+ III (9FA samples) indicating a mix of Hawaiian and Bristol backgrounds in the parental strain that stems from the introgression of the *skeIR1* allele.

To determine chromosome ploidy, we conducted a copy number variant analysis on 50-kb windows of reads mapped to the CB4856 Hawaiian WS288 genome using the delly software [66]. We created a mappability map following established protocols as described (https://github.com/dellytools/delly) using dicey, samtools, and BWA [64, 65, 67]. Chromosomes with an average copy number falling between 1.45 and 2.5 were classified as diploid, those below 1.45 as haploid, and those above 2.5 as triploid. Only diploid chromosomes were considered for the identification of CO sites.

### CO interference analysis

CO interference was measured based on the gamma shape factor of the gamma distribution fitted to the inter-CO distances for genomic data or inter-COSA-1 distances for cytological data [68]. A best-fitting gamma shape factor of 1 corresponds to no interference and higher/smaller shape factor values correspond to increasingly positive/negative interference.

### Immunoblotting

To compare SYP-4 protein levels in *syp-4* mutant animals, 20 young adults (24 h post-L4) were picked into a 30 μl droplet of M9 buffer (22 mM KH_2_PO_4_, 49 mM Na_2_HPO_4_, 86 mM NaCl, 10 mM NH_4_Cl) with 0.1% (v/v) Tween-20 and washed 3 times with M9 buffer with 0.1% (v/v) Tween-20 in 0.2 ml tubes to remove bacteria. Samples were frozen and stored at −80°C for at least 24 h. Samples were thawed and resuspended in 10 μl benzonase buffer (50 mM Tris–HCl pH 8.0, 2 mM MgCl_2_) with ethylenediaminetetraacetic acid (EDTA)-free protease inhibitor (Roche, 11873580001, 1 tablet per 25 ml benzonase buffer). To release chromatin-bound proteins and reduce sample viscosity, samples were sonicated in a water bath at 4°C for five cycles (sonication cycle: 30 s ON/30 s OFF) followed by benzonase treatement at 37°C for at least 1 h with 5 U of benzonase (Millipore, 71205). Sample loading buffer with reducing agent (Invitrogen, NP0007, 4× NuPAGE™ LDS Sample Buffer, and NP0004, 10× NuPAGE™ Sample Reducing Agent) was added to a final 1.5× concentration in a volume of 20 μl. Samples were boiled for 10 min at 70°C, loaded on a 1.0-mm NuPAGE™ 4%–12% (v/v) Bis-Tris precast gel (Invitrogen) and ran in 1× NuPAGE™ MOPS SDS Running Buffer (Invitrogen, NP0001). Sodium dodecyl sulphate–polyacrylamide gel electrophoresis and semi-wet transfer to a methanol-pre-activated polyvinylidene difluoride (PVDF) membrane were performed in an Electrophoresis Mini Gel Tank (Life Technologies, A25977) according to the manufacturer’s instructions. After protein transfer, the PVDF membrane was blocked for 30 min at room temperature in 1× Roche Blocking buffer (Roche, 11096176001) in PBST. The following primary antibodies were used: mouse anti-HA (1:5000, Invitrogen, $\#$A190-138A) and mouse anti-tubulin (1:5000, Sigma–Aldrich, T6199-25UL). Goat anti-mouse-horseradish peroxidase conjugate (1:5000, BioRad, $\#$1706516) was used as secondary antibody. Proteins were detected by chemiluminescence using the Pierce ECL Plus western blotting Substrate kit (Thermo Scientific, 32132) according to the manufacturer’s instructions and images were acquired on a BioRad system. Image processing and quantification were performed using the free software Fiji [54].

### Phosphoproteomic analysis of SYP-4

#### Meiotic nuclei-enriched sample preparation

A population of *syp-4::ha(ie29); gfp::cosa-1(meIs8)* animals was synchronized by bleaching as described in [69]. Around 15 000 synchronized L1-staged worms were plated per 100-mm peptone-enriched nematode growth medium agar plate inoculated with *E. coli* OP-50 and left to grow to young adulthood for 48 h at 20°C. A total of 10–20 plates were prepared. Meiotic nuclei were isolated in Nuclei Purification Buffer [NPB: 25 mM HEPES, pH 7.4, 118 mM NaCl, 48 mM KCl, 2 mM EDTA, 0.5 mM EGTA (ethylene glycol-bis(β-aminoethyl ether)-N,N,N′,N′-tetraacetic acid), 0.2 mM DTT (Dithiothreitol), 0.25 mM spermine, 0.5 mM spermidine, 0.1% (v/v) Tween-20] supplemented with 1× PhosSTOP (Roche, $\#$4906845001) and 2 μg/ml Proteinase Inhibitor (Sigma, $\#$P9599-1ML) based on [70] with some modifications. Meiotic nuclei were released from the gonad tissue by dissection with a razor blade in 5 ml NPB buffer in a 60-mm dish instead of Dounce homogenization. The broken worm solution was transferred to a pre-chilled 15-ml canonical tube and vortexed on high speed for 30 s, followed by 5 min on ice. Then, the solution passed through two 40-μm cell strainers (Fisher Scientific, $\#$07-201-430) and one 20-μm cell strainer (pluriSelect, $\#$43-50020). The isolated nuclei were collected by centrifugation at 2500 × *g* for 5 min at 4°C. The supernatant was removed, the nuclei were resuspended in 200 μl NPB and transferred to a 1.5-ml tube. The meiotic nuclei were spun down at 2500 × *g* for 5 min at 4°C, the supernatant was removed, the nuclei pellet was flash frozen in liquid nitrogen and stored at −80°C until sonication. Three biological replicates were prepared.

#### SYP-4 immunoprecipitation

Isolated meiotic nuclei were resuspended in 500 μl of iCLIP lysis buffer [71] supplemented with 1× PhosSTOP (Roche, $\#$4906845001) and 2 μg/ml Proteinase Inhibitor (Sigma, $\#$P9599-1ML) and incubated on ice for 15 min. Samples were sonicated on a Bioruptor^®^ Pico machine (Diagenode) at 4°C in the ‘low’ setting for five cycles (sonication cycle: 30 s ON; 30 s OFF). Samples were treated with 4 U of TURBO DNase (Fisher Scientific, $\#$10646175) at 37°C for 5 min at 1200 rpm followed by centrifugation at 15 000 × *g* for 15 min at 4°C. The supernatant was transferred to a new tube and 700 μg of protein were incubated with mouse anti-HA antibody-coupled Dynabeads in 500 μl of iCLIP lysis buffer (trypsin-digested samples: 4 μg antibody per 50 μl beads; Fisher Scientific, $\#$26183 and $\#$10515883; chymotrypsin-digested samples: 10 μg antibody per 1.5 mg beads; Fisher Scientific, $\#$26183 and Thermo Fisher, $\#$14311D). The beads were magnetically separated, washed twice with 900 μl High Salt Wash Buffer, once in 500 μl Wash Buffer [71] and then resuspended in 1× NuPAGE™ Sample Loading Buffer (Invitrogen, NP0007) and 1× NuPAGE™ Reducing Agent (Invitrogen, NP0004). Samples were boiled at 95°C for 5 min, loaded on a 1.0-mm NuPAGE™ 4%–12% Bis-Tris precast gel (Invitrogen) and ran in 1× NuPAGE™ MOPS SDS Running Buffer (Invitrogen, NP0001). The gel was stained with Coomassie Staining solution [50% (v/v) EtOH, 10% (v/v) CH_3_COOH, 0.25% (w/v) Coomassie Blue G-250] for 4 h at room temperature, followed by gel destaining with bidistilled water overnight. The gel was further processed by the EMBL Proteomics Core Facility.

#### Mass spectrometry sample preparation

Two biological replicates were digested with trypsin and one with chymotrypsin, since trypsin digestion resulted in low coverage of the C-terminus of SYP-4. Briefly, the bands were cut from the gel and subjected to in-gel digestion with trypsin or chymotrypsin [72]. Peptides were extracted from the gel pieces by sonication for 15 min, followed by centrifugation and supernatant collection. A solution of 50:50 water:acetonitrile, 1% (v/v) formic acid (2× the volume of the gel pieces) was added for a second extraction, and the samples were again sonicated for 15 min, centrifuged and the supernatant pooled with the first extract.

#### Mass spectrometry analysis

The pooled supernatants were subjected to speed vacuum centrifugation. The samples were dissolved in 10 μl of reconstitution buffer [96:4 water: acetonitrile, 1% (v/v) formic acid]. Samples were injected into an UltiMate 3000 nanoRSLC (Dionex, Sunnydale, CA) coupled with a trapping cartridge (μ-Precolumn C18 PepMap 100, 5 μm, 300 μm inner diameter × 5 mm, 100 Å) and an analytical column (nanoEase™M/Z HSS T3 column 75 μm × 250 mm C18, 1.8 μm, 100 Å, Waters). Trapping was carried out with a constant flow of trapping solvent [0.05% (v/v) trifluoroacetic acid in water] at 30 μl/min onto the trapping column for 6 min. Subsequently, peptides were eluted and separated on the analytical column using a gradient composed of Solvent A [(3% (v/v) DMSO (dimethyl sulfoxide), 0.1% (v/v) formic acid in water] and solvent B [3% (v/v) DMSO, 0.1% (v/v) formic acid in acetonitrile] with a constant flow of 0.3 μl/min. During the analytical separation, the percentage of solvent B was stepwise increased from 2% to 4% in 6 min, from 4% to 8% in 1 min, then 8% to 25% in 41 min, and finally from 25% to 40% in another 5 min. The outlet of the analytical column was coupled directly to an Orbitrap Fusion Lumos (Thermo Scientific, SanJose) mass spectrometer using the nanoFlex source. The peptides were introduced into the Orbitrap Fusion Lumos via a Pico-Tip Emitter 360 μm outer diameter × 20 μm inner diameter; 10 μm tip (New Objective) and an applied spray voltage of 2.4 kV, the instrument was operated in positive mode. The capillary temperature was set at 275°C. Full mass scans were acquired for a mass range of 375–1200 m/z in profile mode in the orbitrap with a resolution of 120 000. The filling time was set to a maximum of 50 ms with a limitation of 4e5 ions. The instrument was operated in data-dependent acquisition mode, and LC-MS/MS (liquid chromatography tandem mass spectrometry) scans were acquired in the Orbitrap with a resolution of 30 000, a fill time of up to 86 ms and a limitation of 2e5 ions (AGC target). A normalized collision energy of 34 was applied. MS2 data was acquired in profile mode.

#### Identification of phosphorylated sites in SYP-4

Acquired data were processed by IsobarQuant [73] and Mascot (v2.2.07) was used for protein identification. Data were searched against the UniProt *C. elegans* proteome database containing common contaminants, reversed sequences and the sequences of the proteins of interest. The data were searched with the following modifications: carbamidomethyl (C; fixed modification), acetyl (N-term), oxidation (M), and phospho (STY) (variable modifications). The mass error tolerance for the full scan MS spectra was set to 10 ppm and for the LC-MS/MS spectra to 0.02 Da. A maximum of two missed cleavages was allowed. For protein identification, a minimum of two unique peptides with a peptide length of at least seven amino acids and a false discovery rate below 0.01 were required on the peptide and protein level. The mass spectrometry proteomics data were deposited to the ProteomeXchange Consortium via the PRIDE [74] partner repository with the dataset identifier PXD058372.

### Statistics

All statistical analysis were performed in R. Differences between different strains in embryonic lethality, incidence of male progeny, length of synapsis zone, coefficient of variation of COSA-1 foci intensity and ZHP-3 loading were tested using the nonparametric Mann–Whitney *U* two-sided test from the R Stats package (v4.3.2). Differences in SYP-4 protein levels quantified by western blot were statistically assessed using the nonparametric Wilcoxon Signed-Sum two-sided paired test available in the R Stats package (v4.3.2). Statistical significance between SYP-4 sensitivity to 1,6-hexanediol was tested using a linear mixed-effects model, with genotype as a fixed effect and gonads from individual animals as a random effect. This test allows us to compare hexanediol sensitivity between genotypes across the entire pachytene region while accounting for the fact that measurements within each gonad are not independent. A paired two-sided Student’s *t*-test was used (R Stats package v4.3.2) to assess whether the automated identification of COSA-1 using SpotMAX was statistically different from the manual identification of COSA-1 in the same nucleus. Statistical analysis of the number of COSA-1 foci, number of DAPI-staining bodies, and number of ring-shaped DAPI-staining bodies in different strains was performed using a Gamma–Poisson generalized linear model from the bioconductor glmGamPoi package [75], which is less sensitive to sample size differences compared to other tests. When >10 comparisons were performed against the same strain, a *P*-value correction was performed using the Benjamini–Hochberg correction method available in the R Stats package (v4.3.2).

## Results

**Figure 1.**
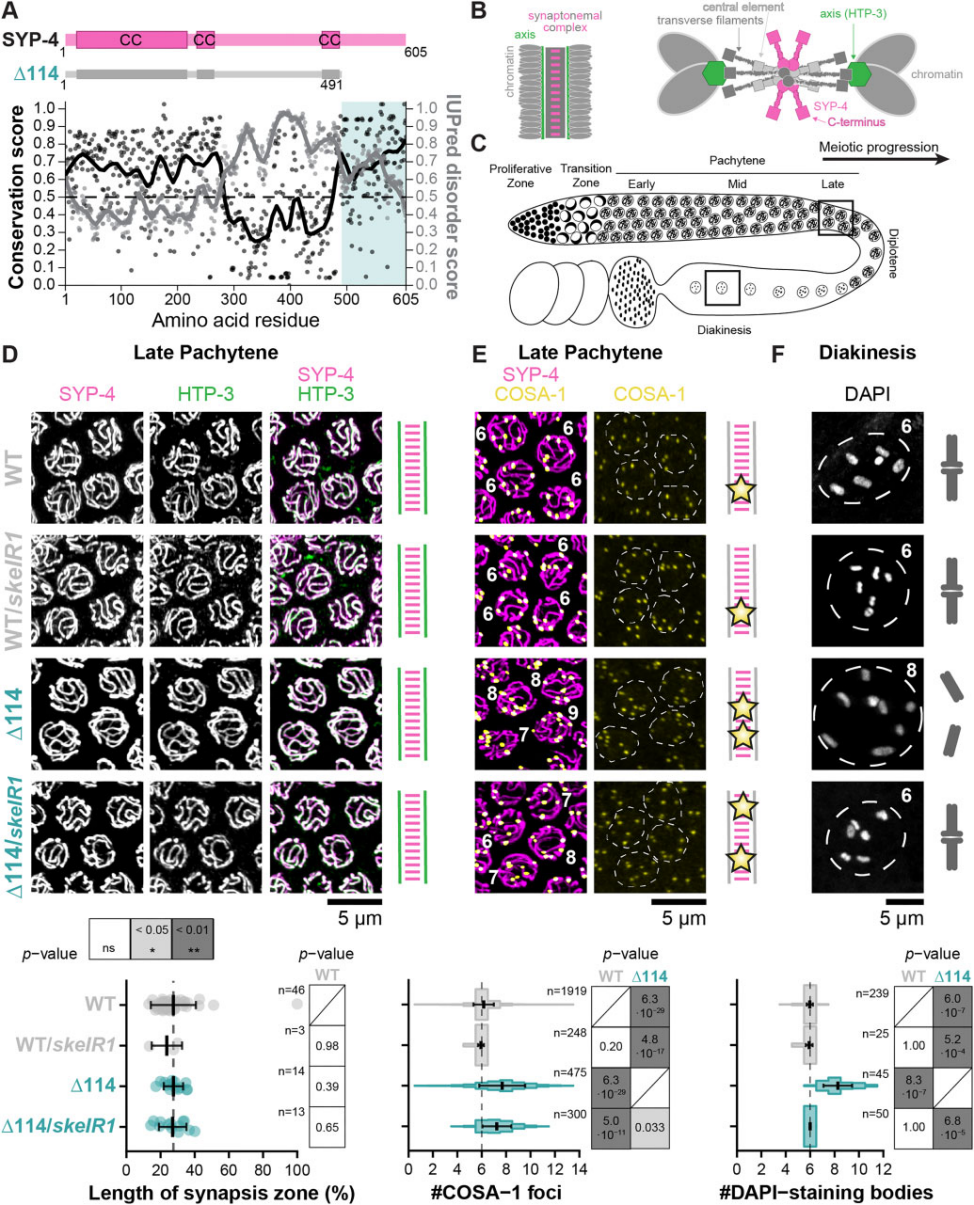
The last 114 amino acids of SYP-4 constitute a conserved domain that is dispensable for synapsis but essential for CO regulation. (**A**) Both the N- and C-termini of *C. elegans* SYP-4 are conserved (black). While the N-terminus is predicted to fold into a CC structure, the C-terminus is predicted to be disordered (grey). To test the functionality of this conserved but disordered C-terminal region, we generated the truncated *syp-4^Δ114^* allele using genome editing. Cartoons of SYP-4 are shown on top. (**B**) Single-molecule localization data predicts the C-terminus of SYP-4 (magenta squares) in the central element of the SC in frontal view (left) and above and below the SC in cross-sectional view (right). Cartoons are based on data from [76] but not drawn to scale. (**C**) The diagram of a *C. elegans* germline depicts the progression through the meiotic prophase I stages along the gonad. Images in panels (D) and (E) were acquired in late pachytene, and images in panel (F) were acquired in diakinesis (black boxes). (**D**) Maximum intensity projections of late pachytene nuclei stained for the HA-tagged SC protein SYP-4 (magenta, left) and the axis protein HTP-3 (green, centre). The merged image is shown on the right. Cartoons illustrate the major finding of complete synapsis across all genotypes with perfect co-localization of axes (green) and SYP-4 (magenta). The time required for synapsis from initiation of synapsis to completion of synapsis is not changed in homozygous *syp-4^Δ114^* or heterozygous balanced *syp-4^Δ114^*/*skeIR1* animals compared to wild-type (WT) animals (bottom). The vertical dashed line shows the average synapsis zone length in WT animals for comparison. Error bars show mean ± standard deviations. The number of analysed gonads is given as *n*. *P*-values were calculated using the Mann–Whitney *U* test and corrected using the Benjamini–Hochberg method. (**E**) Maximum intensity projections of late pachytene nuclei stained for SYP-4::HA (magenta) and the Halo-tagged CO marker COSA-1 (yellow). Cartoons highlight that CO interference may be lost or reduced in homozygous and heterozygous *syp-4^Δ114^* animals where some chromosomes acquire more than one COSA-1 focus (stars). The quantification of COSA-1 foci in late pachytene nuclei (bottom) shows an increase in the number of foci in both homozygous *syp-4^Δ114^* and heterozygous balanced *syp-4^Δ114^*/*skeIR1* animals compared to WT animals. The vertical dashed line at six COSA-1 foci shows the expected number of COSA-1 foci in late pachytene nuclei in WT. Error bars show mean ± standard deviations. The number of nuclei analysed for each genotype is given as *n*. *P*-values were calculated using a Gamma–Poisson generalized linear model and corrected using the Benjamini–Hochberg method. (**F**) Maximum intensity projections of diakinesis nuclei, counterstained with DAPI, show some homolog pairs separating into univalents in *syp-4^Δ114^* animals (cartoons). Quantification is shown at the bottom. The vertical dashed line at 6 DAPI-staining bodies corresponds to the expected number of DAPI-staining bodies per diakinesis nuclei in WT. Error bars show mean ± standard deviations. The number of diakinesis nuclei analysed for each genotype is given as *n*. *P*-values were calculated using a Gamma–Poisson generalized linear model and corrected using the Benjamini–Hochberg method.

We recently showed that a frameshift mutation within the last 19 amino acids combined with the addition of a 3xFlag tag at the C-terminus of SYP-4 constitutes a separation-of-function allele that supports synapsis but causes a severe reduction in CO interference strength during meiosis I [76]. This observation suggested that the C-terminus of SYP-4 is involved in the regulation of CO formation. To understand the role of SYP-4 in this process, we first analysed the amino acid sequence conservation of SYP-4 proteins within the *Caenorhabditis* genus (Fig. 1A). We found conserved regions at the N- and C-terminus of SYP-4. The conserved N-terminus of SYP-4 is predicted to form CC domains that likely interact with other SYP proteins within the SC [16, 19]. By contrast, the conserved C-terminus of SYP-4 is predicted to be largely disordered and its conservation suggests that it may have an important role (Fig. 1A). Notably, our previous single-molecule localization data suggested that the C-terminus of SYP-4 is not embedded within the SC but protrudes above and below the SC in *C. elegans* and is thus accessible to interact with other factors (Fig. 1B) [76].

### The C-terminus of SYP-4 is dispensable for synapsis

To understand the role of the C-terminus of SYP-4 in meiosis, we generated a C-terminally truncated SYP-4 allele, in which the last 114 amino acids were deleted (Fig. 1A, Δ114). In *C. elegans*, the fidelity of chromosome segregation during the meiotic divisions can readily be assessed by quantifying the embryonic lethality and incidence of male progeny: mutations that impair the segregation of chromosomes in meiosis will produce aneuploid progeny, giving rise to high embryonic lethality if any of the autosomes is missegregated or male offspring if the X chromosome is missegregated. Mutated *syp-4^Δ114^* animals showed both high lethality and high incidence of males compared to *syp-4^wt^* animals (Supplementary Fig. S1, 90.5 ± 3.0% versus −2.4 ± 6.1% and 39.7 ± 10.5% versus 0.1 ± 0.2%, respectively; Supplementary Table S5), suggesting that chromosomes were missegregated during meiosis. The increase in embryonic lethality and male progeny in *syp-4^Δ114^* animals was fully rescued in heterozygous *syp-4^Δ114^*/*skeIR1* animals that carry a wild-type *syp-4* allele and resemble *syp-4^wt^*/*skeIR1* animals with around 25% embryonic lethality due to the segregation of the lethal allele in the balancer chromosome (Supplementary Fig. S1, 25.2 ± 8.1% versus. 24.8 ± 5.7% and 0.4 ± 0.6% versus. 0%, respectively; Supplementary Table S5). We created the *skeIR1* balancer for *syp-4* consisting of a lethal gene deletion because the *syp-4^Δ114^* allele was unstable in the presence of the established *hT2* balancer chromosome carrying a large translocation [77] (Supplementary Fig. S2 and Supplementary Table S5).

Mutations in genes constituting the SC typically cause defects in the assembly of the SC, which can drastically reduce the fertility of such animals [14–20, 78]. We therefore tested whether the reduced fertility of *syp-4^Δ114^* animals was caused by defects in SC assembly. To this end, we assessed the co-localization of an axis protein, HTP-3, and the SC component SYP-4 during pachytene. In both, wild-type and *syp-4^Δ114^* animals, axes and SC were perfectly co-localized along the entire length of chromosomes in pachytene suggesting that the *syp-4^Δ114^* allele supports complete synapsis (Fig. 1C and D). We also measured the duration of synapsis. In *C. elegans*, the position of meiotic nuclei within the germline indicates their meiotic stage, as the nuclei move through the germline as they progress through meiosis [79] (Fig. 1C). Therefore, we measured the length of the zone from the first appearance of linear chromosome axes until all chromosomes in a row of nuclei are fully synapsed as a proxy for the duration of synapsis (synapsis zone). The length of this synapsis zone in *syp-4^Δ114^* animals was indistinguishable from wild-type animals (Fig. 1D, 27.5 ± 5.7% versus 27.5 ± 13.2%; Supplementary Fig. S3A). Measuring the expression levels and loading of SYP-4 on the SC using western blotting and quantitative imaging, respectively, (Supplementary Fig. S3B–D) revealed that SYP-4^Δ114^ is more abundant than wild-type SYP-4, and the timing of SYP-4 loading on the chromosome axes is not altered in absence of the C-terminus of SYP-4 in *syp-4^Δ114^* animals (Supplementary Fig. S3B–D). In *C. elegans*, the loading of SC components is interdependent [14–16, 18–20]. Consequently, other SC components should also load in a timely manner and may be more abundant on *syp-4^Δ114^* SCs compared to WT SCs. Indeed, the transverse filament protein SYP-5 exhibited complete synapsis, loaded in a timely manner and was also more abundant on *syp-4^Δ114^* SCs than on WT SCs (Supplementary Fig. S4). These results show that the C-terminus of SYP-4 is dispensable for the assembly and maintenance of the SC but is required for other meiotic processes.

### The C-terminus of SYP-4 regulates CO patterning

The SC is not only essential for maintaining the pairing of homologous chromosomes but also for CO formation [23–26, 76]. In *C. elegans*, the CO factor COSA-1 acts as a cytological marker for designated CO sites in late pachytene [35]. We visualized COSA-1 using an endogenously engineered HaloTag, and quantified the number of COSA-1 foci per nucleus in late pachytene (Fig. 1E). We automated the quantification of COSA-1 foci by integrating automated nucleus segmentation [56] with spot identification using SpotMAX [58]. A comparison between our automated analysis and manual quantifications for 481 nuclei representative of the dataset demonstrated that the automated pipeline yielded results consistent with manual counts (Supplementary Fig. S5). Using the automated quantification of COSA-1 foci, we found that the number of foci per nucleus was elevated from 6.2 ± 0.8 in wild-type to 7.7 ± 1.9 in *syp-4^Δ114^* animals (Fig. 1E). Interestingly, the number of COSA-1 foci was also elevated in heterozygous *syp-4^Δ114^*/*skeIR1* animals (7.2 ± 1.2). The similarity in the number of COSA-1 foci between heterozygous *syp-4^Δ114^*/*skeIR1* and homozygous *syp-4^Δ114^* animals contrasts with the observed rescue of elevated embryonic lethality and male progeny in the heterozygotes (Supplementary Fig. S1 and Supplementary Table S5). Therefore, the increase in COSA-1 foci is insufficient to explain the substantial fertility defects observed in *syp-4^Δ114^* animals. This observation matches previous findings that, while additional COs can lead to chromosome missegregation during meiosis, most chromosomes with surplus COs segregate correctly [7].

While COSA-1 reliably marks designated CO sites in late pachytene in *C. elegans* [35], such designated sites must then progress to form chiasmata to establish the bivalent structure in diakinesis. In wild-type animals, the presence of six bright COSA-1 foci correlates with the formation of six chiasmata, which organize the six pairs of chromosomes into six bivalents observed as 6.0 ± 0.3 DAPI-staining bodies in diakinesis (Fig. 1C and F). Similarly, heterozygous *syp-4^wt^*/*skeIR1* and *syp-4^Δ114^*/*skeIR1* animals exhibited six DAPI-staining bodies (Fig. 1F, 5.9 ± 0.3 and 6.0 ± 0.0, respectively), indicating the establishment of at least one chiasma per homologous chromosome pair. However, in *syp-4^Δ114^* animals, we observed 8.3 ± 1.2 DAPI-staining bodies suggesting that on average only four chromosomes form bivalents, while two pairs of homologs remain as four univalents (Fig. 1F). This finding suggests that either not all designated COs mature into chiasmata in *syp-4^Δ114^* animals or that some chromosomes acquire multiple COs while others lack COs entirely. We observed that both *syp-4^Δ114^* and *syp-4^Δ114^*/*skeIR1* animals display ring-shaped bivalents, indicating the presence of two chiasmata on these chromosomes [80] suggesting that some chromosomes may receive multiple COs (Supplementary Fig. S6A and B).

**Figure 2.**
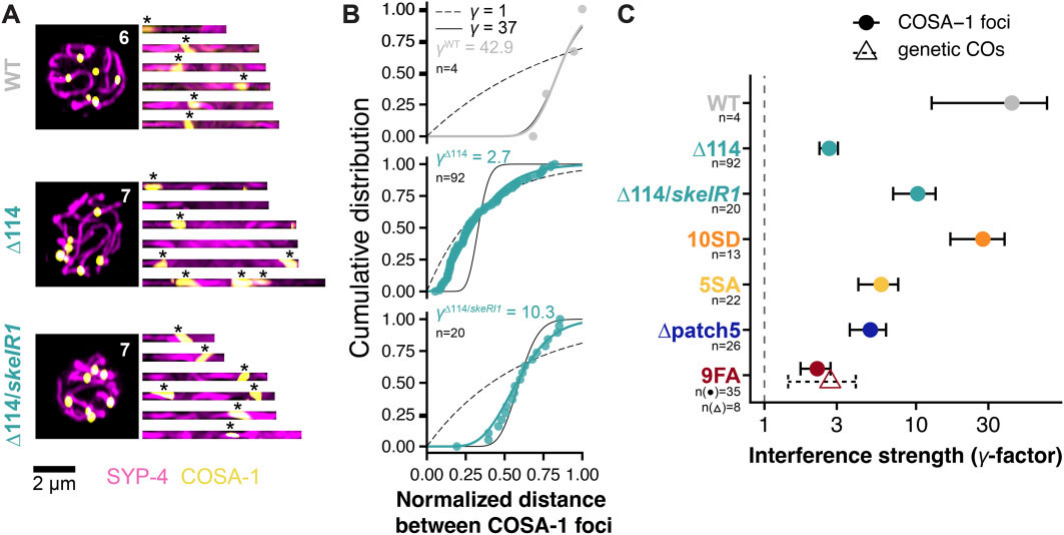
CO interference is attenuated in presence of the *syp-4^Δ114^* allele. (**A**) The number of COSA-1 foci per SC is tightly regulated to exactly one focus per SC in WT animals. This regulation is lost in *syp-4^Δ114^* animals, which have SCs with multiple or no COSA-1 foci, and reduced in *syp-4^Δ114^*/*skeIR1* animals, which have SCs with one or two COSA-1 foci. Maximum intensity projections of representative late pachytene nuclei stained for HA-tagged SYP-4 (magenta) and the Halo-tagged COSA-1 (yellow) are shown on the left, individual straightened SCs are shown on the right. The positions of COSA-1 foci along the chromosomes are marked by asterisks (*). (**B**) Fitting a gamma distribution (solid coloured line) to the cumulative distribution function of normalized inter-COSA-1 distances (circles) in WT (top), *syp-4^Δ114^* (centre), and *syp-4^Δ114^*/*skeIR1* (bottom) indicated that CO interference is reduced in homozygous and heterozygous *syp-4^Δ114^* animals with a γ shape factor of 2.7 and 10.3, respectively, compared to a γ factor of 42.9 in WT animals. Black lines show fits for no interference (γ = 1, dashed line), and expected wild-type interference (γ = 37, solid line [26]). The number of distances between foci is given by *n*. (**C**) CO interference strength is reduced in all *syp-4* mutant animals with elevated numbers of COSA-1 foci as quantified by the γ shape factor as shown in panel (B). The number of distances between foci/COs used to assess interference strength are given by *n*. Error bars show estimated standard errors.

To further characterize the defects in CO regulation in *syp-4^Δ114^* animals, we traced single chromosomes within late pachytene nuclei and quantified the number of COSA-1 foci on each chromosome (Fig. 2A). Our analysis revealed that 32% corresponding to approximately two out of six chromosomes were devoid of COSA-1 foci in *syp-4^Δ114^* animals, while all chromosomes had COSA-1 foci in *syp-4^Δ114^*/*skeIR1* and WT animals (Supplementary Fig. S7A), mirroring the numbers of bivalents formed (Fig. 1F). Additionally, >33% and 19% of chromosomes in *syp-4^Δ114^* and *syp-4^Δ114^*/*skeIR1* late pachytene nuclei, respectively, displayed multiple COSA-1 foci suggesting that interference may be attenuated in presence of the *syp-4^Δ114^* allele (Supplementary Fig. S7A). To assess the strength of CO interference within these chromosomes, we fitted a gamma distribution to the normalized inter-COSA-1 focus distances to estimate the shape parameter (γ) (Fig. 2B). This shape factor serves as a relative measure of CO interference strength, where a γ value of 1 denotes no interference (dashed black line) and higher γ shape parameters indicate stronger interference [11, 68, 81]. In our analysis, we included four wild-type nuclei, each containing a rare occurrence of one chromosome bearing two COSA-1 foci as a proxy for wild-type interference. While these WT chromosomes exhibited robust CO interference (γ = 42.9), consistent with prior studies (solid black line) [26], *syp-4^Δ114^* animals displayed a significantly reduced interference with a γ = 2.7 (Fig. 2B and C). The interference strength of heterozygous *syp-4^Δ114^*/*skeIR1* animals was also reduced but remains stronger than in homozygous *syp-4^Δ114^* animals (Fig. 2B and C; γ = 10.3).

Defects in CO formation are frequently linked with elevated and prolonged levels of RAD-51 foci that indicate the presence of unrepaired DSBs [82]. Indeed, *syp-4^Δ114^* animals also showed elevated and prolonged levels of RAD-51 foci (Supplementary Fig. S8) indicating that the C-terminus of SYP-4 is dispensable for activating the CO assurance checkpoint consistent with earlier data suggesting that this checkpoint is dependent on the chromosome axis [83].

### Putative phosphorylation sites within the C-terminus of SYP-4 do not play a major role in CO patterning

**Figure 3.**
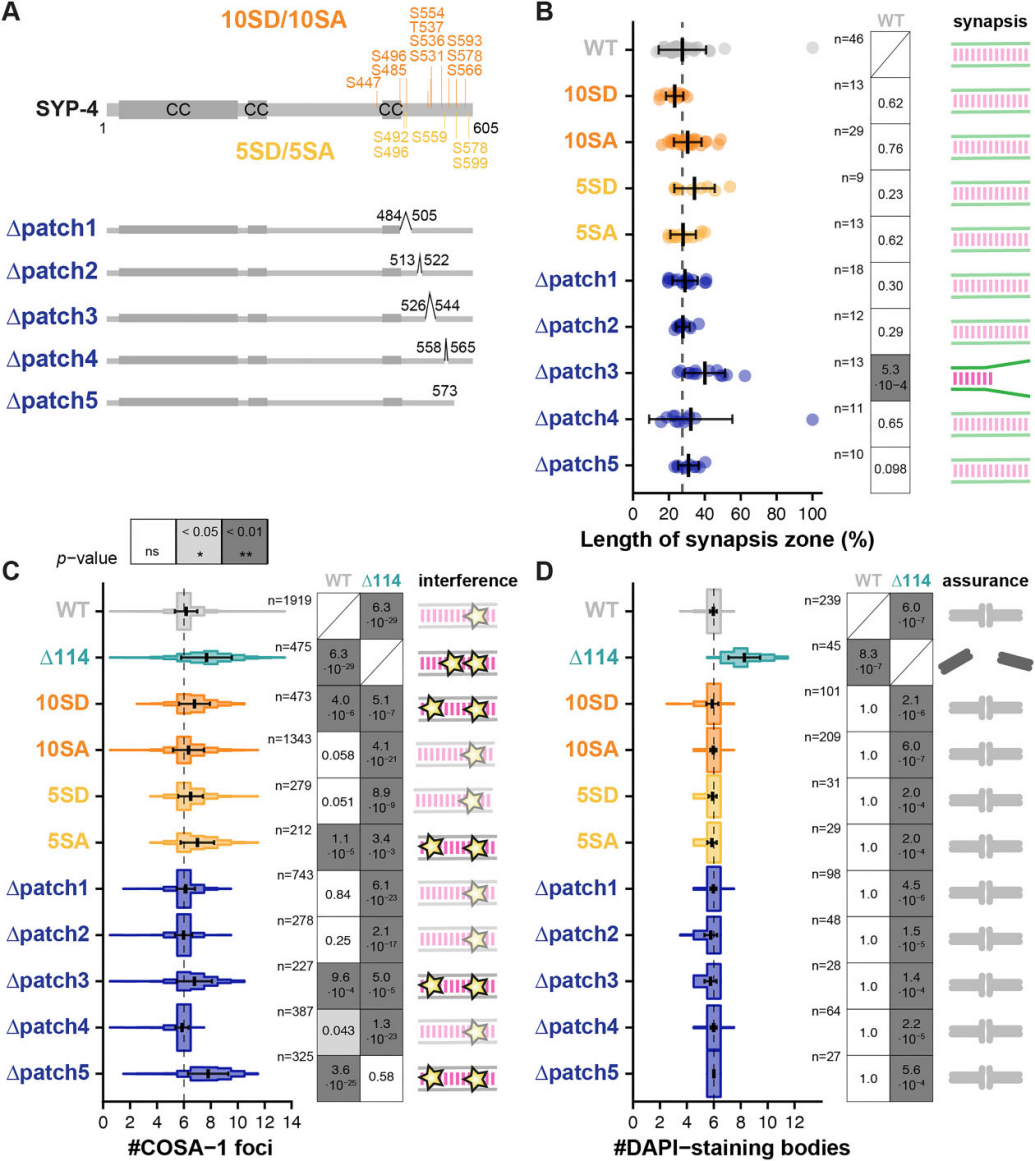
Phosphorylation of the C-terminus of SYP-4 and the last 32 amino acids of SYP-4 modulate CO interference but are dispensable for CO assurance. (**A**) Diagram of SYP-4 indicating the serine/threonine residues substituted by aspartate residues in SYP-4^10SD^ and SYP-4^5SD^ phosphomimetic, or by alanine residues in SYP-4^10SA^ and SYP-4^5SA^ phosphodead mutants (top), and the short internal/terminal deletions within the C-terminus of SYP-4 (bottom). CC corresponds to predicted CC domains. (**B**) The length of the synapsis zone is unaffected in all SYP-4 phosphomutants and almost all short deletions compared to WT animals but it is extended in *syp-4^Δpatch3^* animals as indicated by the cartoons (right). The vertical dashed line corresponds to the average synapsis zone length observed in WT animals. Error bars show mean ± standard deviations. The number of analysed gonads is given as *n*. *P*-values were calculated using the Mann–Whitney *U* test and corrected using the Benjamini–Hochberg method. (**C**) The quantification of COSA-1 foci in late pachytene nuclei shows an increase in the number of foci for *syp-4^10SD^* and *syp-4^5SA^* phosphomutants, *syp-4^Δpatch3^* and *syp-4^Δpatch5^* animals compared to WT animals. Moreover, the observed increase of COSA-1 foci in *syp-4^Δpatch5^* animals is similar to the increase observed in *syp-4^Δ114^* animals. The vertical dashed line at 6 COSA-1 foci corresponds to the expected number of COSA-1 foci per nucleus in WT. Cartoons depict the likely decrease in interference observed for some of the mutants. Error bars show mean ± standard deviations. The number of nuclei analysed for each genotype is given as *n*. *P*-values were calculated using a Gamma–Poisson generalized linear model and corrected using the Benjamini–Hochberg method. (**D**) Quantification of DAPI-staining bodies in diakinesis nuclei indicates the presence of 6 bivalents in most of the quantified nuclei in phosphomutant, short deletion, and WT animals. Cartoons depict the robust bivalent formation in all mutants. Error bars show mean ± standard deviations. The number of diakinesis nuclei analysed for each genotype is given as *n*. *P*-values were calculated using a Gamma–Poisson generalized linear model and corrected using the Benjamini–Hochberg method.

Phosphorylations play a critical role in regulating many aspects during meiotic progression [84]. In *C. elegans*, phosphorylation of residues S269 and S447 in the middle of SYP-4 regulate DSB formation and the choice of the recombination pathway, respectively [85, 86]. Consequently, we hypothesized that the C-terminus of SYP-4 might also undergo phosphorylations to control CO formation. To identify potential phosphorylation sites in the C-terminus of SYP-4 systematically, we conducted mass-spectrometry analysis of SYP-4 purified from young adults (Supplementary Table S7). We identified four potential sites: S447, S485, S496, and S554. Notably, phosphorylation of S447 was previously linked to directing DSB repair toward homologous recombination, thereby preventing repair via the nonhomologous end joining pathway [86]. However, mutating S447 alone did not affect CO interference and assurance, with each chromosome still acquiring exactly one CO in both phosphodead and phosphomimetic S447 mutants [86]. We therefore explored whether multiple phosphorylations of the C-terminus of SYP-4 might collaborate to robustly regulate CO numbers. To test this hypothesis, we mutated all four identified sites from our mass-spectrometry analysis, along with six additional Ser/Thr residues that were not covered, or were under-represented, in our mass-spectrometry analysis (Supplementary Table S7), resulting in the *syp-4^10SD^*/*syp-4^10SA^* phosphomimetic/phosphodead mutants (Fig. 3A, top). Both *syp-4^10SD^* and *syp-4^10SA^* mutants showed reduced fertility with a small increase in embryonic lethality and higher numbers of male progeny (Supplementary Fig. S1, 8.5 ± 4.7% and 5.9 ± 16.0%, 2.8 ± 1.3% and 1.1 ± 0.8%, respectively; Supplementary Table S5) indicating that some aspects of meiosis may be impaired. However, both phosphomimetic and phosphodead *syp-4^10SD^* and *syp-4^10SA^* animals exhibited normal synapsis (Fig. 3B and Supplementary Figs S3A and S9A) and displayed only mild or no defects in CO regulation, characterized by an increase in COSA-1 foci from 6.2 ± 0.8 in wild-type animals to 6.8 ± 1.2 and 6.3 ± 1.2 in *syp-4^10SD^* and *syp-4^10SA^* animals, respectively (Fig. 3C and Supplementary Fig. S9B). The increase in the number of COSA-1 foci in *syp-4^10SD^* animals is associated with a decrease in interference strength, although it remains stronger than in *syp-4^Δ114^* animals (Fig. 2C and Supplementary Fig. S7). CO assurance was also not affected in *syp-4^10SD^* or *syp-4^10SA^* animals and all chromosomes received at least one CO giving rise to six DAPI-staining bodies (Fig. 3D and Supplementary Fig. S9C).

In parallel, we performed an *in silico* analysis using the Eukaryotic Linear Motif (ELM) resource to predict motifs in the C-terminus of SYP-4 [87]. This analysis revealed five potential sites that may bind BRC-1 when phosphorylated. Notably, BRC-1 localizes to the SC and is implicated in regulating the choice of pathways during meiotic DSB repair [88–92]. To investigate whether the putative BRC-1 binding sites within the C-terminus of SYP-4 contribute to CO regulation, we generated phosphomimetic (*syp-4^5SD^*) and phosphodead (*syp-4^5SA^*) mutants (Fig. 3A). Both mutants exhibited almost normal fertility (Supplementary Fig. S1 and Supplementary Table S5) and normal SC assembly and maintenance (Fig. 3B and Supplementary Figs S3A and S9A). Consistent with their largely normal fertility, both *syp-4^5SD^* and *syp-4^5SA^* animals also showed robust CO regulation, although *syp-4^5SA^* animals displayed an increase in the number of designated CO sites marked with Halo-tagged COSA-1 and an associated reduction in CO interference strength compared to wild-type (Fig. 3C and Supplementary Fig. S9B, 7.0 ± 1.2 versus 6.2 ± 0.8; Fig. 2C and Supplementary Fig. S7B and C, γ = 5.9 versus γ = 42.9). The number of DAPI-staining bodies remained unaffected by both mutations (Fig. 3D and Supplementary Fig. S9C).

Together, these findings indicate that phosphorylations of the C-terminus of SYP-4 are dispensable for synapsis and CO assurance but influence the distribution of COSA-1 foci, and thus potentially the recombination landscape in *C. elegans*, as previously proposed [85, 86]. Mutations preventing or mimicking the phosphorylation of potential BRC-1 motifs weaken interference supporting previous research indicating that BRC-1 monitors and modulates meiotic recombination [88–92]. However, these mutations did not recapitulate the strong loss of CO interference and assurance observed in *syp-4^Δ114^* animals.

### The last 32 amino acids of SYP-4 ensure robust CO interference

To investigate whether specific regions are required for regulating CO formation in *C. elegans*, we identified five patches within the C-terminus of SYP-4 that are conserved across the *Caenorhabditis* genus (Fig. 3A, bottom). Deletion of any of these patches resulted in viable offspring (Supplementary Fig. S1 and Supplementary Table S5). The embryonic lethality and male progeny of *syp-4^Δpatch1^*, *syp-4^Δpatch2^*, *syp-4^Δpatch3^*, and *syp-4^Δpatch4^* animals closely resembled wild-type animals, but *syp-4^Δpatch5^* animals exhibited significantly higher embryonic lethality and a higher percentage of male progeny (Supplementary Fig. S1, 38.0 ± 6.9% and 5.3 ± 2.1%, respectively; Supplementary Table S5, respectively), albeit lower than in *syp-4^Δ114^* animals. All animals successfully formed the SC (Supplementary Fig. S9A), and the length of the synapsis zone resembled its length in wild-type animals for four of the five strains carrying the small deletions. However, *syp-4^Δpatch3^* animals exhibited an extension of the synapsis zone length compared to wild-type animals (Fig. 3B and Supplementary Fig. S3A, 40.0 ± 11.3% versus 27.5 ± 13.2%). Given that the deletion of the last 114 amino acids of SYP-4 in *syp-4^Δ114^* animals does not cause synapsis defects, we speculate that the extension of the synapsis zone in *syp-4^Δpatch3^* animals may result from defective folding of SYP-4 and/or reduced protein stability, rather than patch3 being essential for SC assembly.

We further assessed whether deleting short stretches in the C-terminus of SYP-4 affects CO regulation by quantifying COSA-1 foci in late pachytene. While *syp-4^Δpatch1^*, *syp-4^Δpatch2^*, and *syp-4^Δpatch4^* animals displayed an average of six COSA-1 foci, similar to wild-type, *syp-4^Δpatch3^* and *syp-4^Δpatch5^* animals exhibited an increase to 6.8 ± 1.3 and 7.8 ± 1.5 foci, respectively (Fig. 3C and Supplementary Fig. S9B). The increase in the number of COSA-1 foci observed in *syp-4^Δpatch3^* animals likely stems from delayed SC assembly, consistent with previous results for other partial *syp* mutants [25, 26, 93]. However, the increase in *syp-4^Δpatch5^* animals mirrors the defects seen in *syp-4^Δ114^* animals (Fig. 3C). Despite these variations in the number of COSA-1 foci, all animals with deletions in the C-terminus of SYP-4 exhibited six DAPI-staining bodies in diakinesis, indicating complete CO assurance (Fig. 3D and Supplementary Fig. S9C).

Together, these findings suggest that the last 32 amino acids constituting patch5 are essential for restricting the number of designated CO sites to exactly six sites per nucleus. These results are also consistent with our previous data showing that a frameshift mutation affecting the last 19 amino acids in conjunction with the insertion of a 3xFlag epitope tag (*syp-4^CmutFlag^*) severely impairs CO interference but not assurance [76].

### Conserved phenylalanines within the C-terminus of SYP-4 are involved in CO patterning

**Figure 4.**
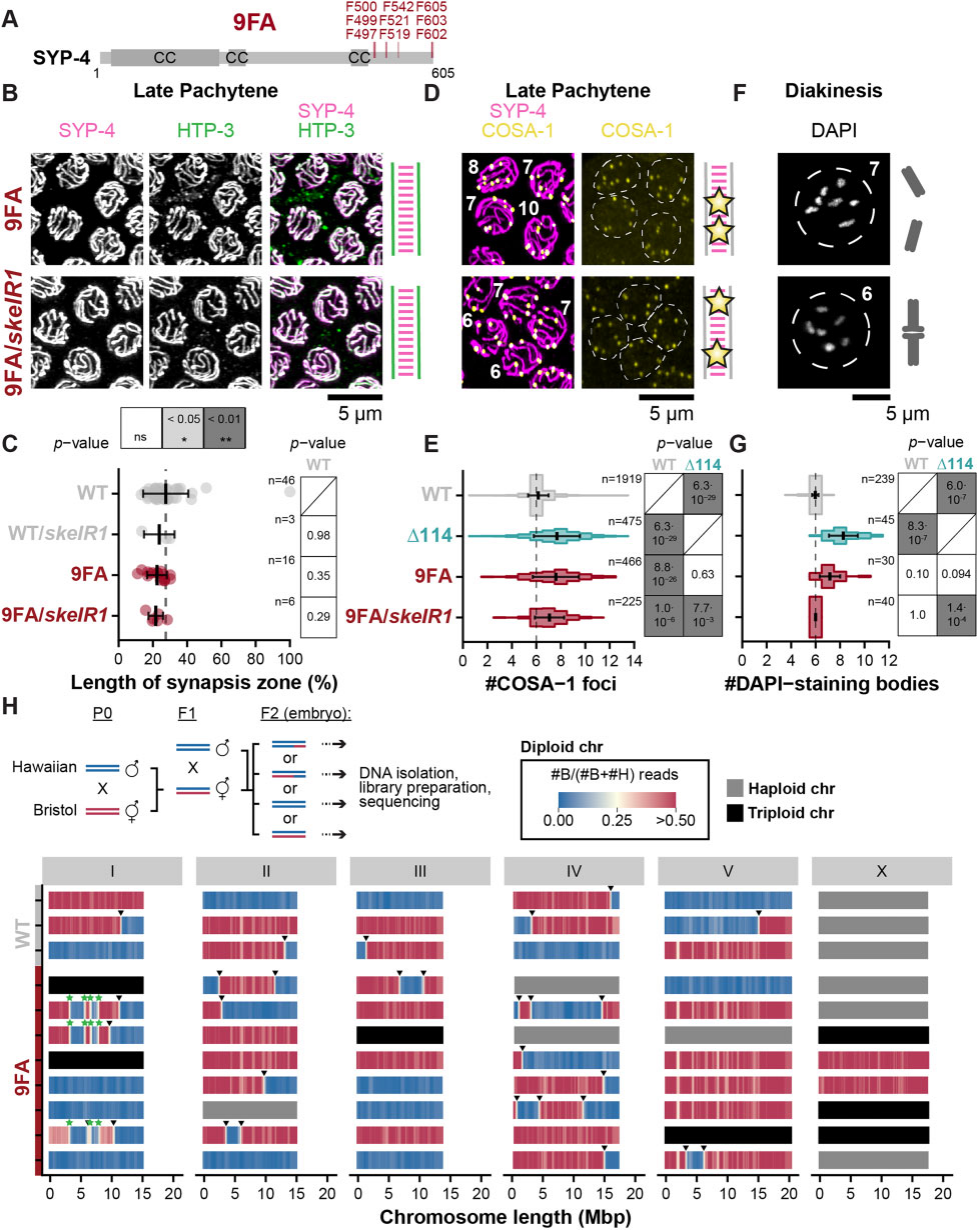
The C-terminus of SYP-4 regulates CO formation via conserved phenylalanine residues. (**A**) Diagram of SYP-4 indicating the phenylalanine residues substituted by alanine residues in *syp-4^9FA^* animals. CC corresponds to predicted CC domains. (**B**) Maximum intensity projections of late pachytene nuclei stained for HA-tagged SYP-4 (magenta, left) and the axis protein HTP-3 (green, centre). The merged image is shown on the right. (**C**) The synapsis zone length is not altered in homozygous *syp-4^9FA^* or heterozygous balanced *syp-4^9FA^*/*skeIR1* animals compared to WT animals. The vertical dashed line corresponds to the average synapsis zone length observed in WT animals. Error bars show mean ± standard deviations. *n* denotes the number of gonads analysed for each genotype. *P*-values were calculated using the Mann–Whitney *U* test and corrected using the Benjamini–Hochberg method. (**D**) Maximum intensity projections of late pachytene nuclei stained for SYP-4::HA (magenta) and the Halo-tagged CO marker COSA-1 (yellow). (**E**) Quantification of COSA-1 foci in late pachytene nuclei shows an increase in the number of foci in both homozygous *syp-4^9FA^* and heterozygous balanced *syp-4^9FA^*/*skeIR1* animals compared to WT animals suggesting a semi-dominant effect of the *syp-4^9FA^* allele. The vertical dashed line at 6 COSA-1 foci shows the expected number of COSA-1 foci in late pachytene nuclei in WT. Error bars show mean ± standard deviations. The number of nuclei analysed for each genotype is given as *n*. *P*-values were calculated using a Gamma–Poisson generalized linear model and corrected using the Benjamini–Hochberg method. (**F**) Maximum intensity projections of diakinesis nuclei counterstained with DAPI. (**G**) Quantification of DAPI-staining bodies in diakinesis nuclei shows an increase in the number of DAPI-staining bodies in homozygous *syp-4^9FA^* animals compared to WT indicating the presence of univalents, while the number of DAPI-staining bodies is not increased in heterozygous balanced *syp-4^9FA^*/*skeIR1* animals. Error bars show mean ± standard deviations. The number of diakinesis nuclei analysed for each genotype is given as *n*. *P*-values were calculated using a Gamma–Poisson generalized linear model and corrected using the Benjamini–Hochberg method. (**H**) Genetic mapping of CO sites confirms loss of assurance and interference in *syp-4^9FA^* animals. A schematic representation of the experimental strategy followed to genetically map CO sites is shown on top. Chromosome tracks show the ratio between Bristol-specific reads and the total number of specific reads along the chromosomes. A ratio of zero (blue) corresponds to homozygous regions derived from the Hawaiian background, whereas a ratio of 0.5 or more (red) corresponds to heterozygous regions containing both Hawaiian- and Bristol-specific sequences. The transition from homozygous (blue) to heterozygous (red) regions corresponds to CO sites (arrow heads). Chromosomes that were found to contain a single copy (haploid, grey) or three copies (triploid, black) based on the average copy number per chromosome were not considered for the detection of CO sites. Recombination scars from the introgression of the *skeIR1* balancer on chromosome I of *syp-4^9FA^* animals are marked by green stars. We sequenced three wild-type and 8 *syp-4^9FA^* embryos. Note that *syp-4^9FA^* but not wild-type animals included a *HaloTag::cosa-1* allele and a C-terminal HA-tag for *syp-4*. However, the addition of these tags did not alter the phenotype of WT animals (Supplementary Table S5).

Since neither phosphorylations nor single conserved patches in the C-terminus of SYP-4 could explain the severe defects in CO regulation observed in *syp-4^Δ114^* animals, we investigated other characteristics of the C-terminus of SYP-4 to determine its role in CO regulation. Analysing the amino acid composition revealed that the C-terminus of SYP-4 is enriched in phenylalanines: while Phe constitutes only 4% of all residues in the full-length protein, Phe makes up 14% of the C-terminus of SYP-4. Nine of the sixteen Phe in the C-terminus of SYP-4 are located in motifs that are conserved across the *Caenorhabditis* genus and resemble nematode-specific LIR-motifs that typically mediate the interaction with Atg8-related proteins in autophagy [87]. To test whether these Phe have a specific function during meiosis in SYP-4, we generated a mutant, *syp-4^9FA^*, replacing the nine Phe in LIR-motifs by alanines (Fig. 4A). *syp-4^9FA^* exhibited very high embryonic lethality and produced a high number of male progeny (Supplementary Fig. S1, 91.8 ± 6.4% and 20.1 ± 13.7%, respectively; Supplementary Table S5) that resembled the brood counts of *syp-4^Δ114^* animals. Like *syp-4^Δ114^* animals, *syp-4^9FA^* animals were also proficient for synapsis and exhibited higher levels of SYP-4^9FA^ and SYP-5 along the chromosome axes (Fig. 4B and C, and Supplementary Figs S3A and D and S4), although the expression level of SYP-4^9FA^ was lower than wild-type SYP-4 and SYP-4^Δ114^ (Supplementary Fig. S3B and C). In accordance with the strong reduction in fertility, *syp-4^9FA^* animals also displayed an increase in the number of COSA-1 foci to 7.6 ± 1.9 compared to 6.2 ± 0.8 in wild-type animals, which resembles the increase in *syp-4^Δ114^* animals (Fig. 4D and E). We also observed an increase in the number of COSA-1 foci in balanced heterozygous *syp-4^9FA^*/*skeIR1* animals to 7.1 ± 1.2 foci suggesting that the *syp-4^9FA^* mutation is also semi-dominant (Fig. 4D and E). Similar to *syp-4^Δ114^* animals, *syp-4^9FA^* animals but not balanced *syp-4^9FA^*/*skeIR1* showed an increase in the number of DAPI-staining bodies, suggesting that not all homologs are held together by CO events (Fig. 4F and G, 7.2 ± 0.8). Indeed, COSA-1 foci are not evenly distributed across chromosomes: some chromosomes in *syp-4^9FA^* animals lack any COSA-1 focus, while other chromosomes have more than one focus (Supplementary Fig. S7). Moreover, interference of COSA-1 foci is strongly impaired, similar to what we found for *syp-4^Δ114^* animals (Fig. 2C and Supplementary Fig. S7C). We also observe ring-shaped bivalents in diakinesis in *syp-4^9FA^* animals suggesting the presence of at least two COs on these chromosomes (Supplementary Fig. S6).

Interestingly, the intensity of COSA-1 foci is more variable in *syp-4^Δ114^* and *syp-4^9FA^* animals than in wild-type animals (Supplementary Fig. S10). Since only ‘bright’ COSA-1 foci typically mature into COs [94], we tested whether all COSA-1 foci produce genetic COs in *syp-4^9FA^* animals by mapping recombination events in hybrids of two divergent *C. elegans* strains, Bristol N2 and Hawaiian CB4856, carrying a *syp-4^wt^* or a *syp-4^9FA^* allele using whole genome sequencing (Fig. 4H). We analysed recombination events in backcrossed F2 embryos instead of F2 adults and their progeny due to the high embryonic lethality in *syp-4^9FA^* animals (Fig. 4H). Analysing copy number variations showed that about 50% of the X chromosomes were haploid indicating male embryos, as expected. However, we also found triploid X chromosomes in *syp-4^9FA^* animals indicating that the X chromosome missegregated. We therefore excluded the X chromosome from all further analysis. We also excluded chromosome I from further analysis since we found scars from the introgression of the *skeIR1* balancer from the Hawaiian background in *syp-4^9FA^* animals (Fig. 4H, green stars). In wild-type animals, all autosomes were diploid and we identified six COs in 15 autosomes suggesting that 40% of the chromosomes received a CO (Fig. 4H). In *syp-4^9FA^* animals, 20% of autosomes were aneuploid suggesting that autosomes were also missegregated as already indicated by the increased number of DAPI-staining bodies (Fig. 4H). Similarly, our analysis revealed the presence of multiple COs at variable distances in *syp-4^9FA^* animals. We found on average 0.7 COs corresponding to 1.5 COSA-1 foci per pair of homologous chromosomes (*n* = 26), suggesting that interference is reduced. Furthermore, in cases where multiple COs were detected, the distribution of inter-CO distances resembled the distribution of COSA-1 foci with a γ shape factor of 2.7 (Fig. 2C and Supplementary Fig. S7C). This finding confirms the significant decrease in interference in *syp-4^9FA^* animals compared to wild-type animals.

### The C-terminus of SYP-4 is required to recruit ZHP-3 to the SC

**Figure 5.**
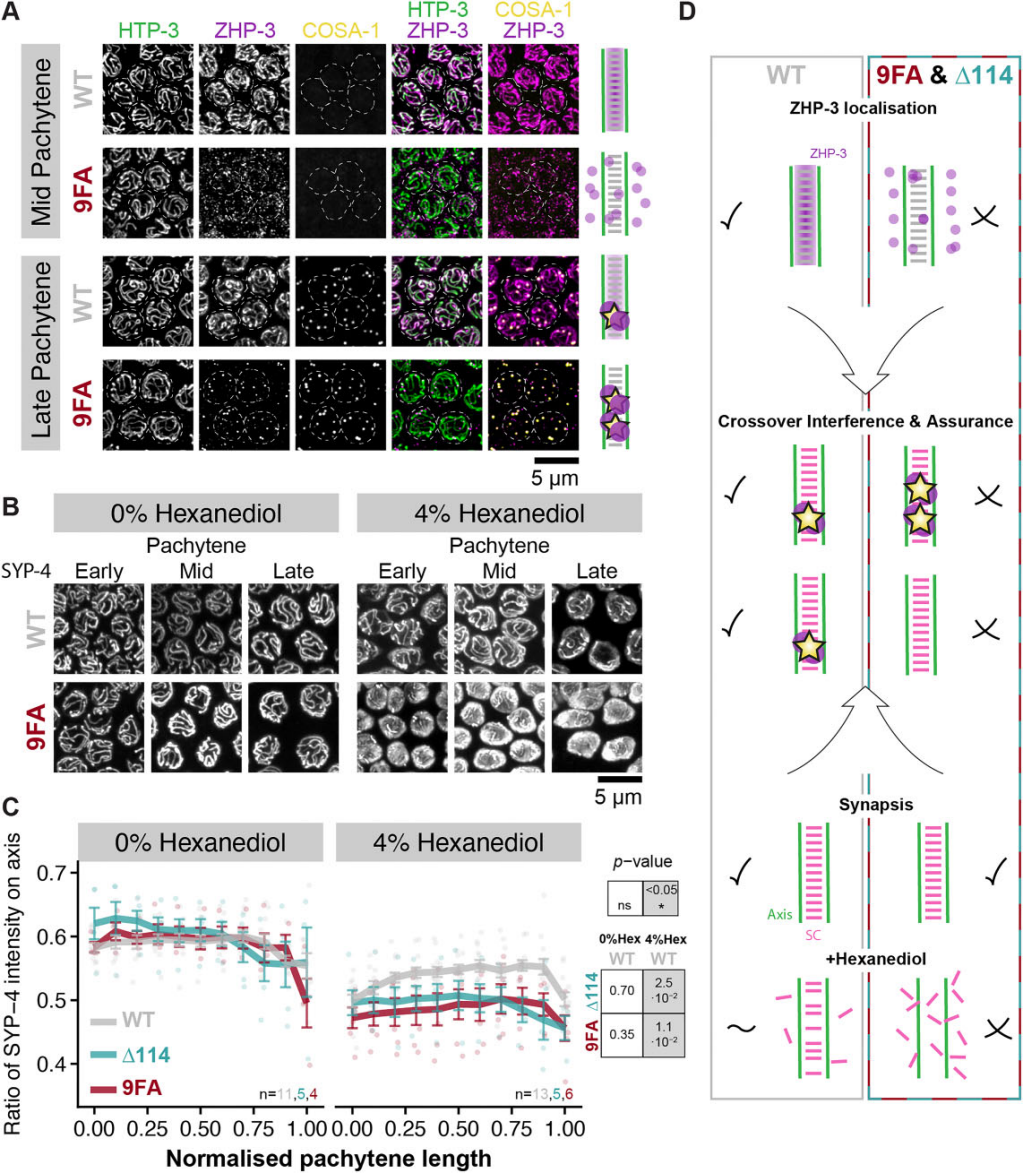
The defects in CO regulation in *syp-4^9FA^* and *syp-4^Δ114^* animals are accompanied by a mislocalization of ZHP-3 and changes in the biophysical properties of the SC. (**A**) Maximum intensity projections of mid and late pachytene nuclei stained for HTP-3 (green), V5-tagged ZHP-3 (magenta), and Halo-tagged COSA-1 (yellow) show that the co-localization of ZHP-3 and SCs is lost in *syp-4^9FA^* animals. However, ZHP-3 co-localizes with COSA-1 in both wild-type and *syp-4^9FA^* animals in late pachytene. Merged images are shown on the right. Nuclei are encircled with a dashed line. (**B**) Maximum intensity projections of early, mid, and late pachytene nuclei from extruded gonads treated with 0% (*w/v*) or 4% (*w/v*) 1,6-hexanediol and stained for SYP-4::HA. (**C**) SCs in *syp-4^9FA^* and *syp-4^Δ114^* animals are more sensitive to 1,6-hexanediol than SCs of WT animals. The ratio between the total SYP-4 intensity on the axes and the total SYP-4 intensity within the nucleus in WT (grey), *syp-4^9FA^* (red), and *syp-4^Δ114^* (cyan) are shown across the pachytene region divided into 11 bins. In untreated pachytene nuclei, the majority of SYP-4 signal is found on the axis (axis/nucleus ratio > 0.5), while SYP-4 is more nucleoplasmic in presence of 4% (w/v) 1,6-hexanediol. The loss of SYP-4 on the axis in presence of hexanediol is higher in *syp-4^9FA^* (*P*-value = 0.011) and *syp-4^Δ114^* animals (*P*-value = 0.025). Error bars show mean ± standard error, and *n* denotes the number of gonads analysed. *P*-values were calculated using a linear mixed model accounting for variations between genotypes and random variations between gonads from different animals (see the ‘Materials and methods’). (**D**) Diagram summarizing the role of nine conserved phenylalanine residues within the C-terminus of SYP-4 in ZHP-3 localization (top) and SC stability (bottom) to regulate crossing-over (centre).

The fact that COs still form but are completely misregulated in *syp-4^Δ114^* and *syp-4^9FA^* animals is reminiscent of the phenotype observed in SC-deficient *A. thaliana* [23, 24, 95]. In both cases, COs occur but they are not regulated through assurance and interference. In *Arabidopsis*, this misregulation is due to the failure of SC assembly, which prevents the recruitment of the E3 ligase HEI10 [23, 24, 31]. Therefore, we investigated whether the HEI10 homolog ZHP-3 is mislocalized in C-terminally mutated *syp-4* animals. ZHP-3 is required for CO formation [32] and co-localizes with SC proteins until late pachytene in wild-type animals [33] (see Fig. 1C for cartoon of meiotic progression in *C. elegans*). However, it is predominantly nucleoplasmic in *syp-4^Δ114^* and *syp-4^9FA^* animals (Fig. 5A and Supplementary Fig. S11). Interestingly, ZHP-3 loading is reduced in *syp-4^Δpatch5^* animals, which exhibit defects in interference (Supplementary Fig. S11). This suggests that proper loading of ZHP-3 is necessary for CO interference. However, in *syp-4^Δ114^*/*skeIR1* heterozygous animals, which also show weakened interference, ZHP-3 loading is similar to WT animals (Supplementary Fig. S11), indicating that any defects may be below our detection limit or that additional mechanisms are required for robust interference.

At the end of pachytene, most ZHP-3 foci co-localize with COSA-1, likely marking CO sites in both wild-type and mutant animals (Fig. 5A and Supplementary Fig. S11A). These results suggest that ZHP-3 localization at recombination sites is independent of the C-terminus of SYP-4. While this association is sufficient for CO formation, its recruitment to the SC appears to be essential for the proper regulation of this process.

The SC has previously been demonstrated to exhibit liquid-like properties [27]. Given that the accumulation of hydrophobic phenylalanines in disordered protein domains has been described to enhance the tendency of such domains to undergo phase separation [96–100], we investigated whether the critical phenylalanines we identified affect the biophysical characteristics of the SC. To do so, we examined the response of SCs from both WT and *syp-4^Δ114^* or *syp-4^9FA^* animals to hexanediol.

In the absence of hexanediol, SC proteins assemble along chromosome axes in both WT and *syp-4^Δ114^* or *syp-4^9FA^* SCs. For all genotypes, the majority of SYP-4 staining is found on the chromosome axes in absence of hexanediol, with both *syp-4* mutant animals showing no difference from WT animals (Fig. 5B and C and Supplementary Fig. S12). However, in the presence of 4% (w/v) hexanediol, the SC partially dissolves into the nucleoplasm (Fig. 5B and C and Supplementary Fig. S12) [27]. Wild-type SYP-4 shows a more stable association with the axes throughout pachytene compared to both SYP-4^Δ114^ and SYP-4^9FA^. This increased sensitivity to hexanediol in the mutant forms is surprising, given that these mutations increase overall SYP protein loading onto the SC (Supplementary Figs S3D and S4). The heightened sensitivity of ‘denser’ SCs in *syp-4^Δ114^* and *syp-4^9FA^* animals indicates altered interactions among SC subunits. Thus, we propose that the phenylalanines located in the C-terminus of SYP-4 play a role in stabilizing the SC, thereby influencing its biophysical properties.

Together, our data demonstrates a role of the disordered C-terminus of the central element protein SYP-4 in meiotic CO regulation, revealing that phenylalanines within this region are essential for recruiting the pro-CO factor ZHP-3 and stabilizing the SC.

## Discussion

Our study revealed that the C-terminus of SYP-4 is dispensable for SC assembly, but it plays a crucial role in controlling CO formation, regulating both CO interference and CO assurance.

Specifically, we demonstrated that removing the C-terminus of SYP-4, or mutating nine phenylalanines within this region, not only alters the physical characteristics of the SC but is also required for the localization of the pro-CO factor and E3 ligase ZHP-3 to the SC. Notably, disordered domains with conserved amino acid sequences, which are found in the C-terminus of SYP-4, were previously classified as ‘constrained disordered domains’ [101]. Such constrained disordered domains are frequently found in domains that simultaneously interact with multiple binding partners. Thus, the C-terminus of SYP-4 may act as a structural hub for regulating CO formation.

The misplacement of ZHP-3 from the SC to the nucleoplasm in both *syp-4^Δ114^* and *syp-4^9FA^* animals is accompanied by significant defects in CO assurance and interference, resembling the CO defects observed in synapsis-deficient *Arabidopsis* [23, 24, 95].

In *Arabidopsis*, this finding prompted the hypothesis that a primary role of the SC is to restrict the movement of the pro-CO factor HEI10, the plant homolog of ZHP-3, to the SC. With restricted movement, recombination events along a single SC must compete for a limited pool of HEI10 molecules, leading to the formation of only a few COs that are spaced far apart—a process termed ‘coarsening’ [30]. Therefore, the absence of synapsis in *Arabidopsis*, or in our case, the failure to recruit ZHP-3 to the SC due to phenylalanine mutations in the C-terminus of SYP-4, may replace the localized coarsening of HEI10/ZHP-3 within the SC by a broader coarsening across the nucleus [31]. This mislocalization of ZHP-3/HEI10 may result in a loss of CO interference and assurance, while maintaining a relatively stable number of COs through CO homeostasis [31]. Therefore, our observation that the loss of CO interference and assurance, but not homeostasis, in *syp-4^Δ114^* and *syp-4^9FA^* animals correlates with a failure to recruit ZHP-3 to the SC is readily explained by the coarsening model. However, it remains unclear whether this model can also explain that a decrease in interference in both *syp-4^Δpatch5^* and heterozygous *syp-4^Δ114^*/*skeIR1* animals is accompanied by decreased ZHP-3-loading along the SC in *syp-4^Δpatch5^* animals but not in heterozygous *syp-4^Δ114^*/*skeIR1* animals. Consequently, additional mechanisms independent of the coarsening of ZHP-3 along the SC may be required for the robust regulation of CO formation.

For example, ZHP-3 may have additional crucial roles at the SC in regulating CO formation that are independent of its predicted coarsening dynamics. Evidence suggests that the localization of ZHP-3 and its heterodimer partner, ZHP-4, is intricately linked to their enzymatic activity, as mutations in their E3 RING finger domains not only abolish CO formation but also prevent their loading along the SC [102, 103]. Therefore, it is plausible that the enzymatic activity of the ZHP-3/ZHP-4 heterodimer may serve additional functions at the SC that are independent of and may precede their coarsening behaviour. Additionally, the biophysical properties of the SC that are affected by the C-terminus of SYP-4 may directly contribute to regulating the distribution of CO events. Such changes could affect binding of CO factors to the SC and/or recombination sites and thus differentially regulate the DNA repair pathway choice.

While the entire disordered C-terminus of SYP-4, or the phenylalanines dispersed within it, are crucial for establishing both CO assurance and interference, we also identified a specific segment within this domain that is essential for ensuring robust CO interference. This segment contains the final 32 amino acids of the C-terminus and is essential for ensuring that CO interference operates across distances longer than individual chromosomes in *C. elegans*. This finding confirms our earlier observations that a mutation in the last 19 amino acids of SYP-4 significantly diminished the strength of CO interference [76].

Surprisingly, despite the abundance of potential phosphorylation sites within the C-terminus of SYP-4, phosphorylations are not a major regulatory mechanism for CO formation but they make CO regulation more resilient. Our findings are thus in agreement with prior data showing that recombination is influenced by SYP-4 phosphorylation, with post-translational modifications within its C-terminus playing a role in determining the recombination pathway and maintaining SC integrity under conditions of elevated DSBs [85, 86]. Thus, post-translational modifications might serve to increase the robustness of CO regulation in *C. elegans*, particularly under challenging circumstances.

The conservation of the SYP-4 C-terminus suggests that its function in regulating CO events may be conserved in other nematodes and maybe even in more distant metazoans. For example, a mutation in the likely disordered C-terminal domain in the mammalian *SIX6OS1* central element component was also linked to defects in CO regulation [104, 105]. These data therefore suggest that the role of the SC, and in particular, of the disordered C-terminus of SYP-4, in regulating CO formation may be conserved across metazoans.

## Supplementary Material

gkaf095_Supplemental_Files

## Data Availability

Sequencing data for recombination mapping are available on the European Nucleotide Archive under accession number PRJEB76871. The mass spectrometry proteomics data were deposited to the ProteomeXchange Consortium via the PRIDE [74] partner repository with the dataset identifier PXD058372. Raw microscopy files are available at https://www.ebi.ac.uk/biostudies/bioimages/studies/S-BIAD1599. All other study data are included in the article and as supplementary information.
