# Supplementary material for: Crossovers are regulated by a conserved and disordered synaptonemal complex domain
Source: Nucleic Acids Res. 2025 Feb 18;53(4):gkaf095. doi: 10.1093/nar/gkaf095 (PMC11833701; doi:10.1093/nar/gkaf095)
Supplement: gkaf095_Supplemental_Files [file gkaf095_supplemental_files.zip › Neves-etal_Supplement2.pdf]

Crossovers are regulated by a conserved and disordered synaptonemal complex domain

Supplemental Information

Ana Rita Rodrigues Neves[1,2], Ivana Čavka[1,2], Tobias Rausch[3,4], Simone Köhler[1,*]

[1] European Molecular Biology Laboratory (EMBL), Cell Biology and Biophysics Unit, Heidelberg, Germany
[2] Collaboration for joint PhD degree between EMBL and Heidelberg University, Faculty of Biosciences, Heidelberg, Germany
[3] European Molecular Biology Laboratory (EMBL), Genome Biology Unit, Heidelberg, Germany.
[4] European Molecular Biology Laboratory (EMBL), GeneCore, Heidelberg, Germany.
[*] To whom correspondence may be addressed. Email: simone.koehler@embl.de

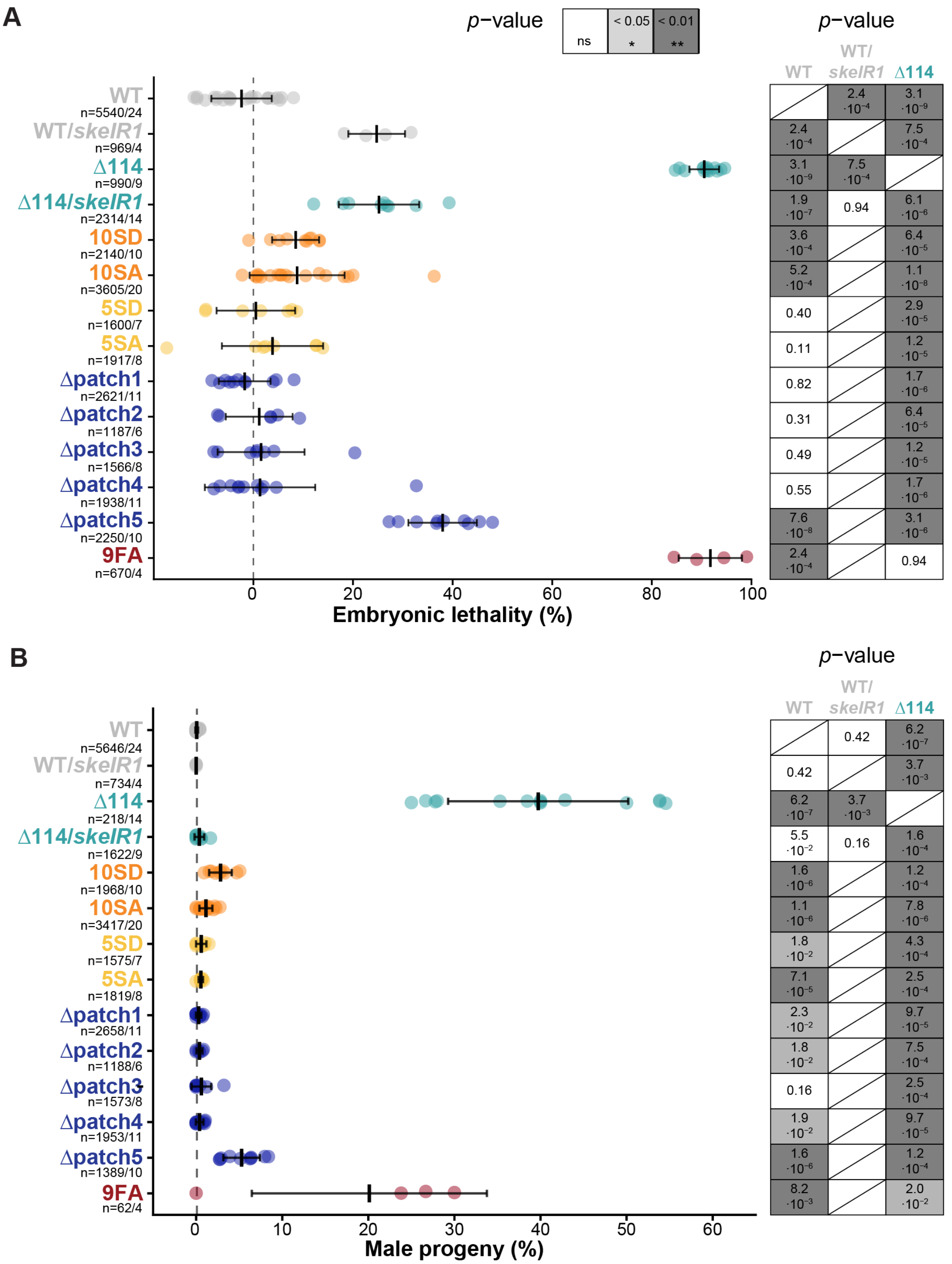

Fig. S1: **Brood counts for strains generated in this study.** (A) The quantification of embryonic lethality in the SYP-4 mutant alleles generated in this study reveals an increased lethality in *syp-4*$^{\Delta 114}$ and *syp-4*^{9FA} animals with more than 90% lethality compared to 0% lethality in WT animals. Embryonic lethality is also increased in Δpatch5, in 10SD and 10SA animals (38%, 8% and 6%, respectively). The heterozygous balanced WT/*skeIR1* and *syp-4*$^{\Delta 114}$/*skeIR1* show a lethality of about 25% as expected. The dashed vertical line at 0% embryonic lethality corresponds to the expected value in WT animals. Error bars show mean ± standard deviations. The total number of counted eggs (E) laid by N animals is indicated for each strain as n=E/N. (B) The incidence of male progeny is significantly increased in the majority of the SYP-4 mutant alleles. This increase is more pronounced in *syp-4*$^{\Delta 114}$, *syp-4*^{9FA} and *syp-4*$^{\Delta patch5}$ animals (40%, 20% and 5%, respectively). The dashed vertical line at 0.1% male progeny corresponds to the expected value in WT animals. Error bars show mean ± standard deviations. The total number of adult progeny (A) from N animals is indicated for each strain as n=A/N. *P*-values were calculated using the Mann-Whitney *U* test and, for comparisons to WT and *syp-4*$^{\Delta 114}$, corrected using the Benjamini-Hochberg method.

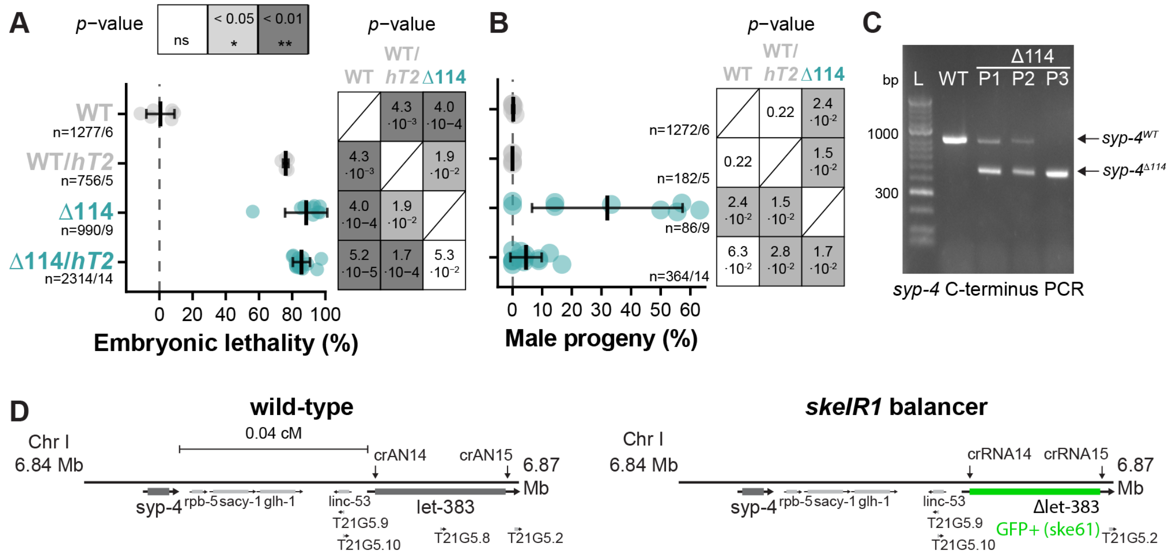

Fig. S2: **The *hT2* balancer chromosome is unstable in *syp-4*$^{\Delta 114}$/*hT2* animals.** (A) The embryonic lethality in heterozygous balanced *syp-4*$^{\Delta 114}$/*hT2* animals is increased compared to the heterozygous balanced WT/*hT2* animals indicating a dominant effect of the *syp-4*$^{\Delta 114}$ allele in presence of the *hT2* balancer. The dashed vertical line at 0% embryonic lethality corresponds to the expected value in WT animals. Error bars show mean ± standard deviations. The total number of counted eggs (E) laid by N animals is indicated for each strain as n=E/N. (B) The incidence of male progeny is increased not only in homozygous *syp-4*$^{\Delta 114}$ animals (32%) but also heterozygous balanced *syp-4*$^{\Delta 114}$/*hT2* animals (5%) although the latter increase is not statistically significant (p-value=0.063). The dashed vertical line at 0.1% male progeny corresponds to the expected value in WT animals. The total number of adult progeny (A) from N animals is indicated for each strain as n=A/N. P-values in A and B were calculated using the Mann-Whitney U test. Error bars show mean ± standard deviations. (C) Representative agarose gel for genotyping *syp-4*^{wt} and *syp-4*$^{\Delta 114}$ alleles in WT animals as well as in non-green supposedly homozygous *syp-4*$^{\Delta 114}$ progeny of heterozygous *syp-4*$^{\Delta 114}$/*hT2* animals from three different plates (P1, P2 and P3) shows the loss of homozygosity of the *syp-4*$^{\Delta 114}$ allele with the incorporation of a *syp-4*^{wt} allele in P1 and P2. (D) The SYP-4-specific balancer, *ske61*, was generated in the CB4856 Hawaiian strain by replacing the *let-383* lethal gene locus with a codon-optimised GFP construct. The diagram depicts the chromosome I region between 6.84-6.78 Mbp containing the *syp-4* and *let-383* gene loci, which are 0.04 cM apart (wild-type is shown on the left). CRISPR RNAs crAN14 and crAN15 (Table S2) were used to replace the *let-383* gene with a GFP transgene.

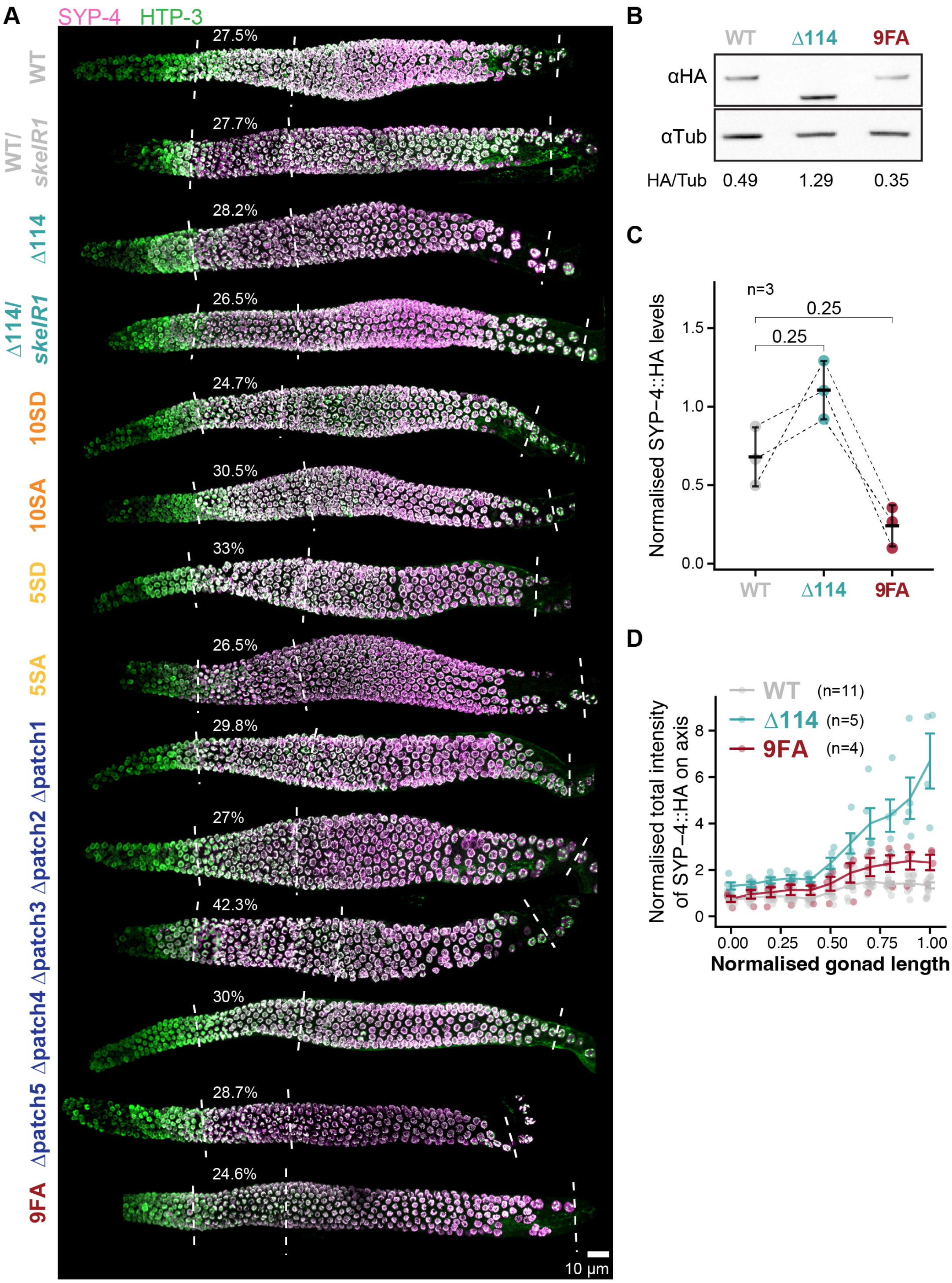

Fig. S3: **Synapsis is normal in *syp-4*$^{\Delta 114}$ and *syp-4*^{9FA} animals.** (A) Representative maximum intensity projections of gonads stained for the HA-tagged SC protein SYP-4 (magenta) and the axis protein HTP-3 (green) used for the quantification of the duration of synapsis. The beginning and end of the synapsis zone and pachytene are delimited by dashed lines. The percentage of synapsis zone length compared to the combined length of synapsis zone and pachytene for each gonad is given. (B) Representative Western blot analysis to quantify the HA-tagged SYP-4 protein levels relative to α-tubulin protein levels in WT, *syp-4*$^{\Delta 114}$ and *syp-4*^{9FA} whole worm lysates. Three Western Blots were analysed for each genotype. (C) Quantification of HA-tagged SYP-4 protein levels from Western blots in WT, *syp-4*$^{\Delta 114}$ and *syp-4*^{9FA} normalised to α-tubulin protein levels show that SYP-4 levels are increased in *syp-4*$^{\Delta 114}$ animals and decreased in *syp-4*^{9FA} animals compared to WT animals but these changes are not statistically significant. Three independent experiments were performed. P-values were calculated using the Wilcoxon Signed-Rank test for two-paired samples. (D) Quantification of SYP-4 loading along the axis from the beginning of transition zone to the end of pachytene shows an increase in the loading of SYP-4 to the SC with meiotic progression in *syp-4*$^{\Delta 114}$ (cyan), *syp-4*^{9FA} (red), and in WT (gray) animals. The amount of protein loaded in the SC is higher in *syp-4*$^{\Delta 114}$ and *syp-4*^{9FA} animals than in WT animals. The length corresponding to the combined transition zone and pachytene region was normalised and divided into 11 bins. The error bars show mean ± standard error. The number of gonads analysed is indicated as n.

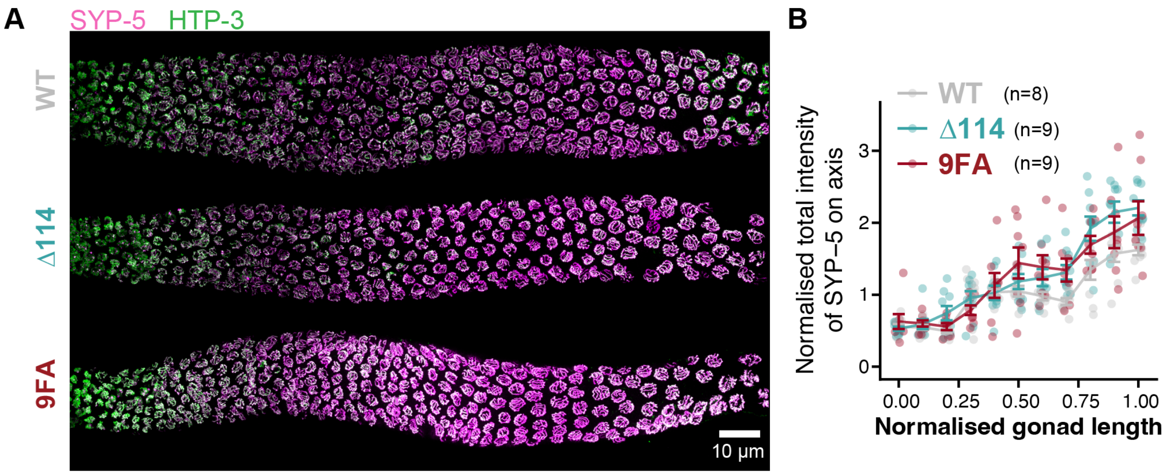

Fig. S4: **Loading of the transverse filament protein SYP-5 is elevated in *syp-4*$^{\Delta 114}$ and *syp-4*^{9FA} animals.** (A) Representative maximum intensity projections of gonads stained for the transverse filament SYP-5 (magenta) and the axis protein HTP-3 (green). SYP-5 co-localises along the entire length of the chromosome axes, indicating that synapsis is complete. (B) The quantification of SYP-5 loading along the axis from the beginning of transition zone to the end of pachytene reveals that both *syp-4*$^{\Delta 114}$ and *syp-4*^{9FA} animals load more SYP-5 than WT animals. The length corresponding to the combined transition zone and pachytene region was normalised and divided into 11 bins. The error bars show mean ± standard error. The number of gonads analysed is indicated as n.

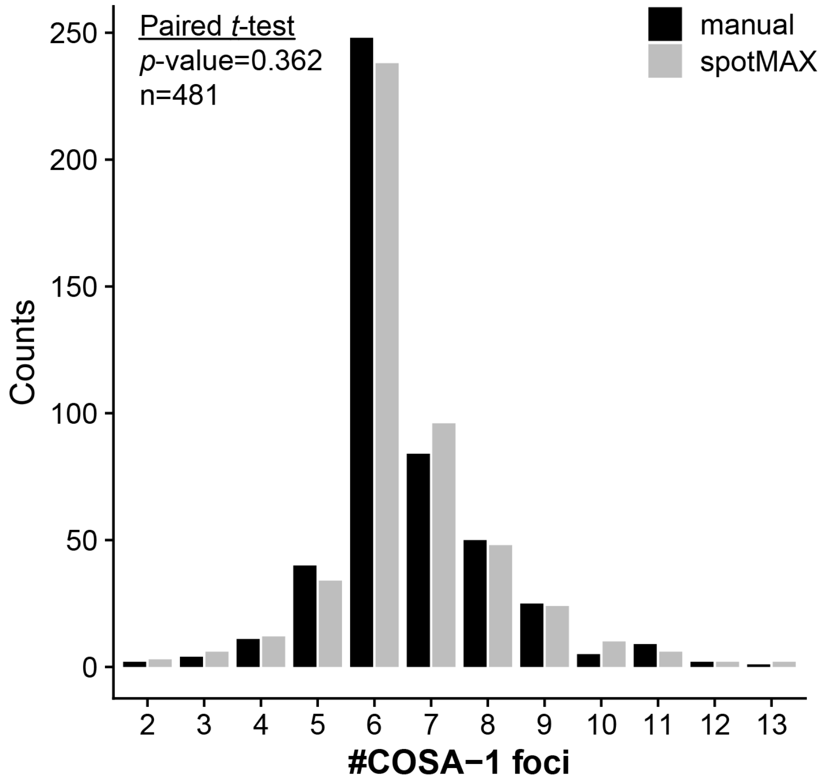

Fig. S5: **SpotMAX automated quantification of COSA-1 foci in 3D images from *C. elegans* fixed gonads is comparable to manual quantification.** Distribution of the number of COSA-1 quantified manually (black) and automatically using SpotMAX (gray) shows no significant difference between the two methods. The same nuclei were quantified by the two approaches. A total number of 481 nuclei originating from different genetic backgrounds used in this study were quantified. No biases related to the genetic background were observed. The *p*-value was calculated using the Student's *t*-test for two-paired samples, and n denotes the number of nuclei analysed

.

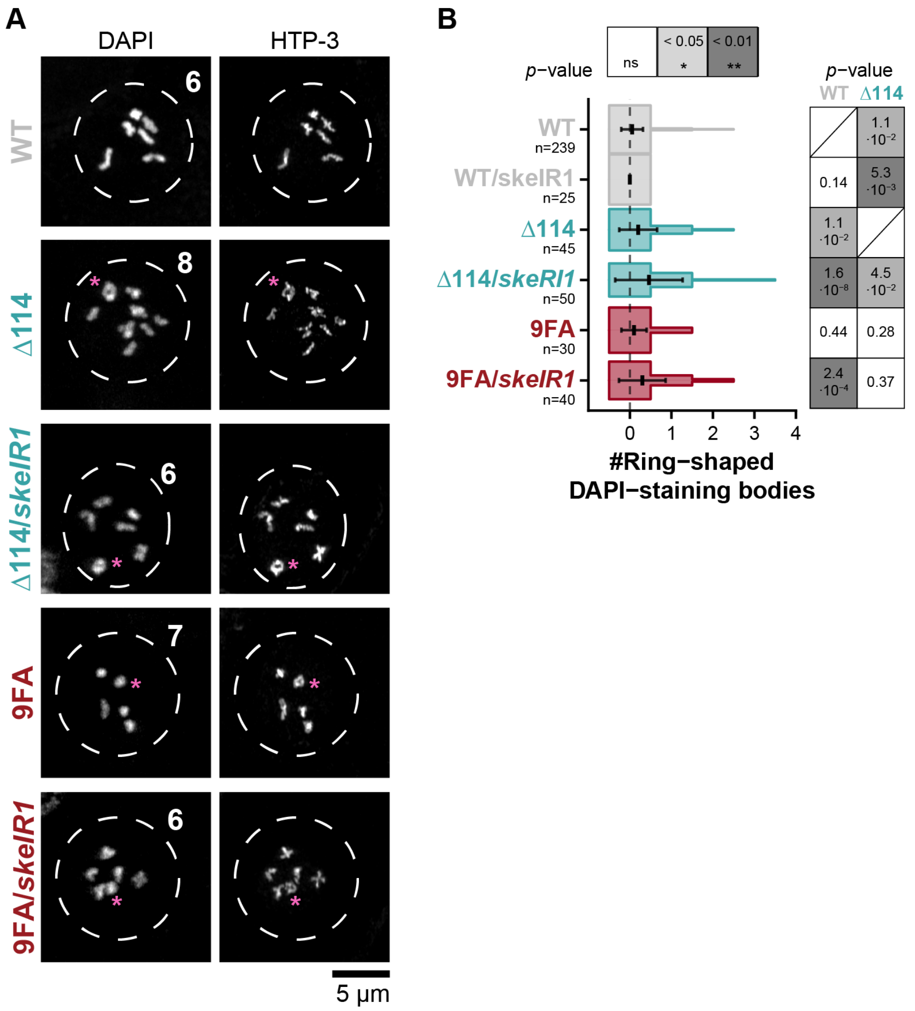

Fig. S6: **Ring-shaped bivalents can be visualised during diakinesis in homozygous as well as heterozygous balanced *syp-4*$^{\Delta 114}$ and *syp-4*^{9FA} animals indicating an increased number of crossovers per bivalent.** (A) Maximum intensity projections of diakinesis nuclei counterstained with DAPI (left) and stained against the axis protein HTP-3 (right). The number of DAPI-staining bodies counted in each nucleus is given. Ring-shaped DAPI-staining bodies are indicated with an asterisk (*). (B) Quantification of number of ring-shaped DAPI-staining bodies reveals ring-shaped bivalents in *syp-4*$^{\Delta 114}$ and *syp-4*^{9FA} mutant alleles in both homozygosity as well as heterozygosity but not wild-type animals supporting the semi-dominant effect suggested by the increased number of COSA-1 foci in these mutants. The number of quantified diakinesis nuclei is given by n. *P*-values were calculated using a Gamma-Poisson generalised linear model.

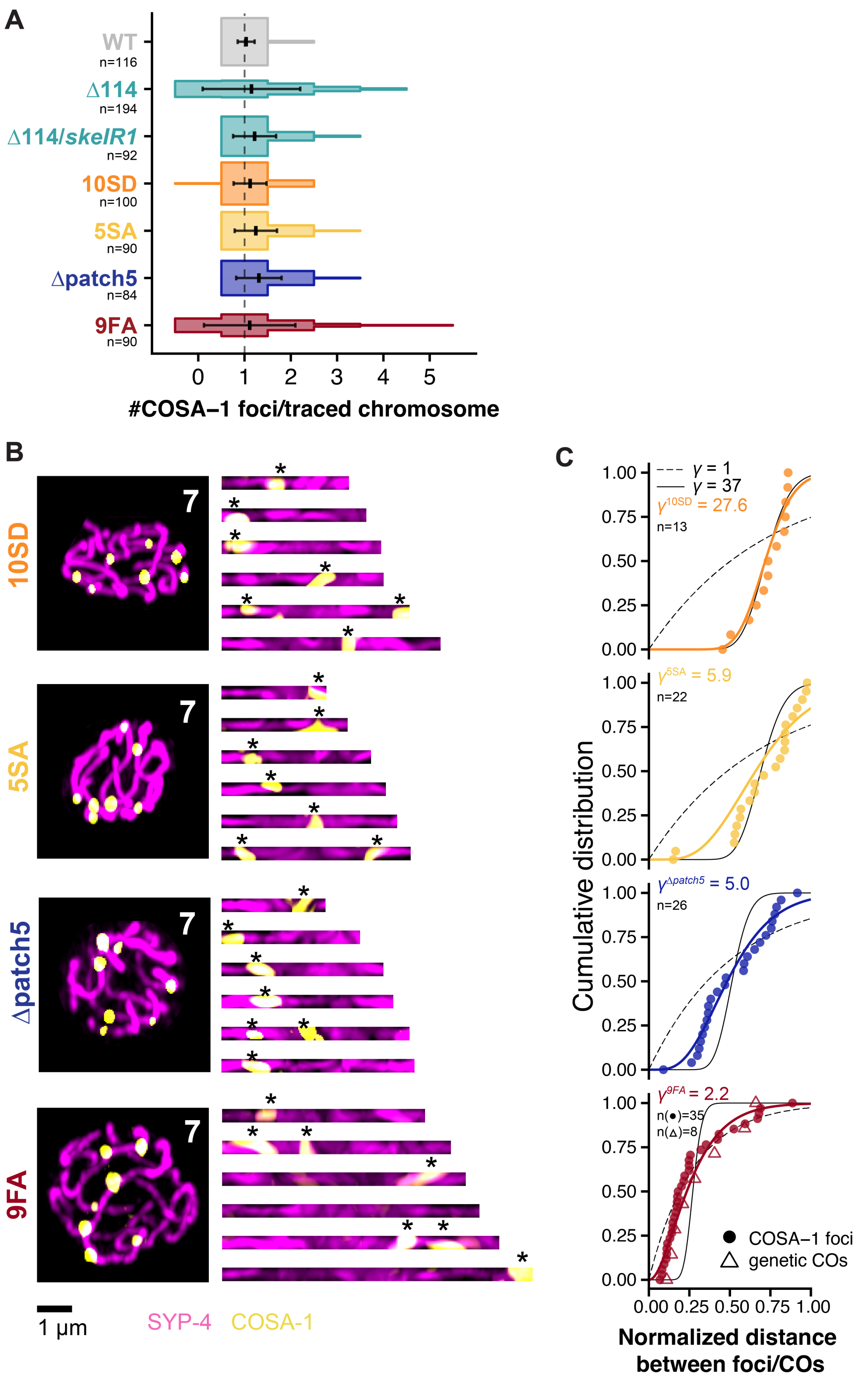

Fig. S7: **Crossover regulation is attenuated by mutations in the C-terminus of SYP-4.** (A) The distribution of the number of COSA-1 foci per traced chromosome in pachytene nuclei shows that 32% and 28% of the chromosomes in *syp-4*$^{\Delta 114}$ and *syp-4*^{9FA}, respectively, have no COSA-1 focus indicating loss of assurance. In contrast, assurance is robust in *syp-4*^{10SD}, *syp-4*^{5SA}, and *syp-4*$^{\Delta patch5}$. At the same time, about 33% of the traced chromosomes in *syp-4*$^{\Delta 114}$ and *syp-4*^{9FA} have more than one COSA-1 focus per chromosome indicating a reduction in crossover interference strength. Likewise, *syp-4*^{10SD}, *syp-4*^{5SA}, and *syp-4*$^{\Delta patch5}$ also increase the number of chromosomes with 2 or more COSA-1 foci (13%, 23% and 29%, respectively). The number of chromosomes analysed is given by n. (B) Maximum intensity projections of representative late pachytene nuclei stained for HA-tagged SYP-4 (magenta) and the Halo-tagged COSA-1 (yellow) are shown on the left, individual straightened SCs are shown on the right. The positions of COSA-1 foci along the chromosomes are marked by asterisks (*). (C) Fitting a gamma distribution (solid coloured line) to the cumulative distribution function of normalised inter-COSA-1 distances (circles) in *syp-4*^{10SD}, *syp-4*^{5SA}, *syp-4*$^{\Delta patch5}$, and *syp-4*^{9FA} (red) indicates that crossover interference is reduced in these animals compared to the expected value of WT animals (γ=37 (26), solid black line). However, only *syp-4*^{9FA} animals show a strong reduction in interference that resembles the expected distribution without interference (γ=1, dashed black line). The severe reduction of crossover interference in *syp-4*^{9FA} was confirmed genetically by the analysis of normalised inter-crossover distances (empty triangles) which have a γ factor of 2.7. The number of distances between foci/COs used to assess interference strength are given by n.

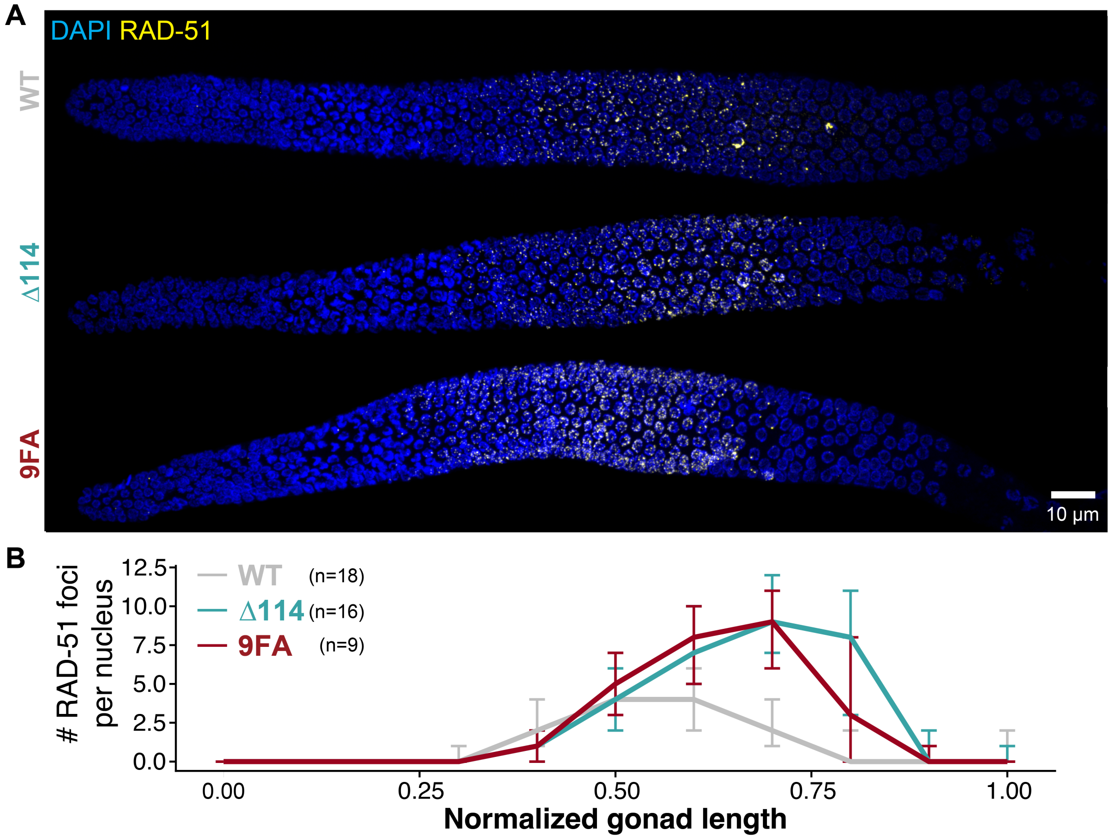

Fig. S8: **The number of RAD-51 foci are elevated in *syp-4*$^{\Delta 114}$ and *syp-4*^{9FA} animals.** (A) Maximum intensity projections of representative gonads stained for DAPI (blue) and V5::RAD-51 (yellow) reveal a prolonged region positive for RAD-51 foci in *syp-4*$^{\Delta 114}$ and *syp-4*^{9FA} animals compared to WT animals. (B) The quantification reveals that the number of RAD-51 foci per nucleus is higher in *syp-4*$^{\Delta 114}$ and *syp-4*^{9FA} animals than in WT animals. The number of gonads analysed for each genotype is given by n.

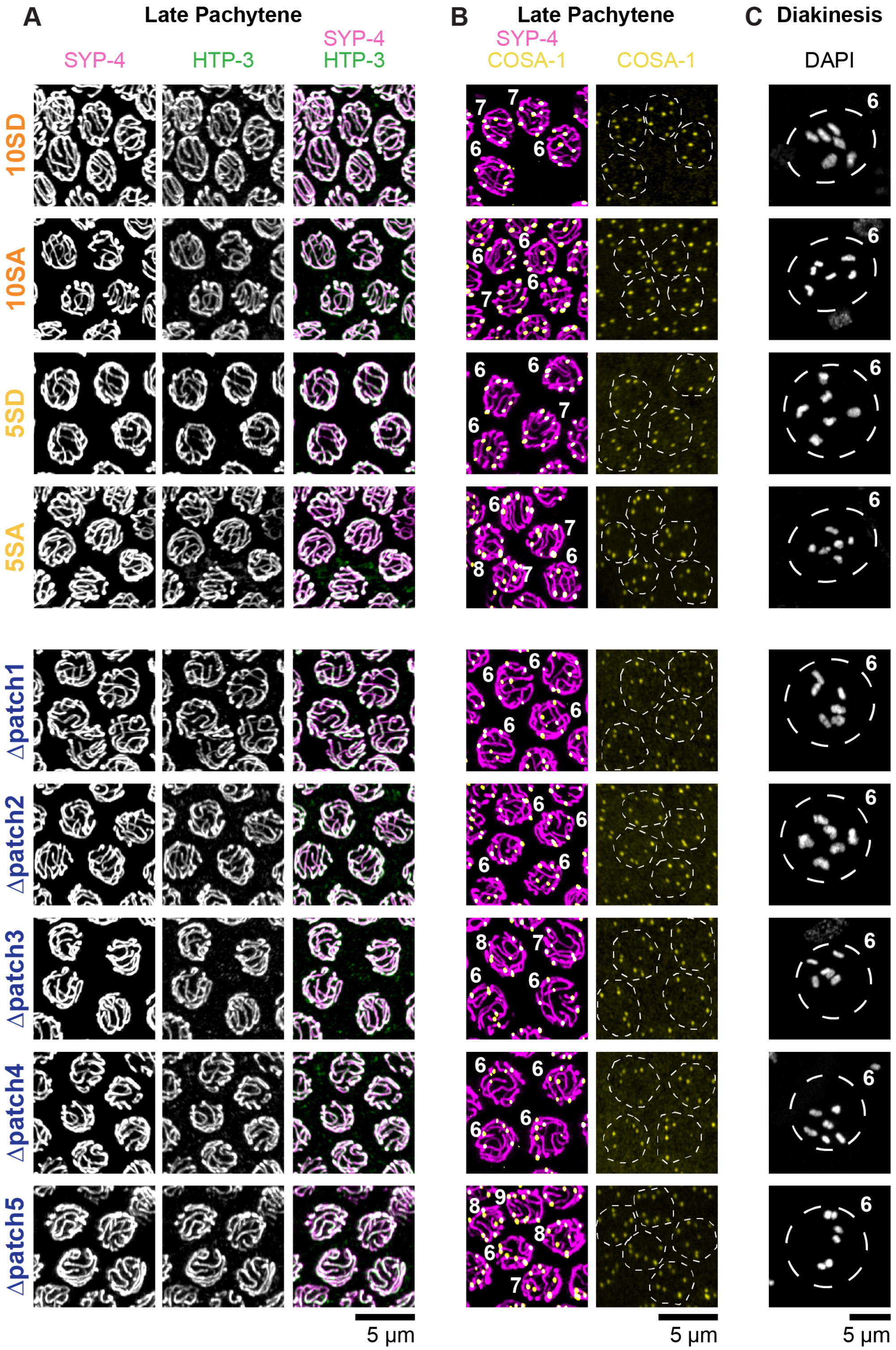

Fig. S9: **Crossover regulation is attenuated by mutations in the C-terminus of SYP-4.** (A) Maximum intensity projections of late pachytene nuclei stained for the HA-tagged SC protein SYP-4 (magenta, left) and the axis protein HTP-3 (green, center). The merged image is shown on the right. (B) Maximum intensity projections of late pachytene nuclei stained for SYP-4::HA (magenta) and Halo::COSA-1 (yellow). (C) Maximum intensity projections of diakinesis nuclei counterstained with DAPI.

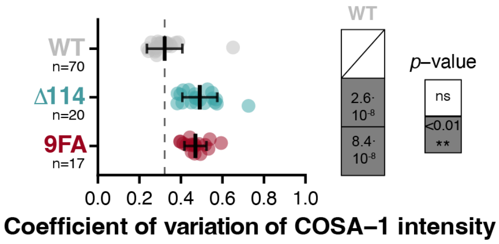

Fig. S10: **COSA-1 foci intensities are variable in *syp-4*$^{\Delta 114}$ and *syp-4*^{9FA} animals.** The variability of fluorescence intensities of Halo-tagged COSA-1 foci measured by the coefficient of variation (s.d./mean) is higher in *syp-4*$^{\Delta 114}$ and *syp-4*^{9FA} animals than WT animals. Error bars show mean ± standard deviations. *P*-values were calculated using the Mann-Whitney *U* test, and n denotes the number of gonads analysed.

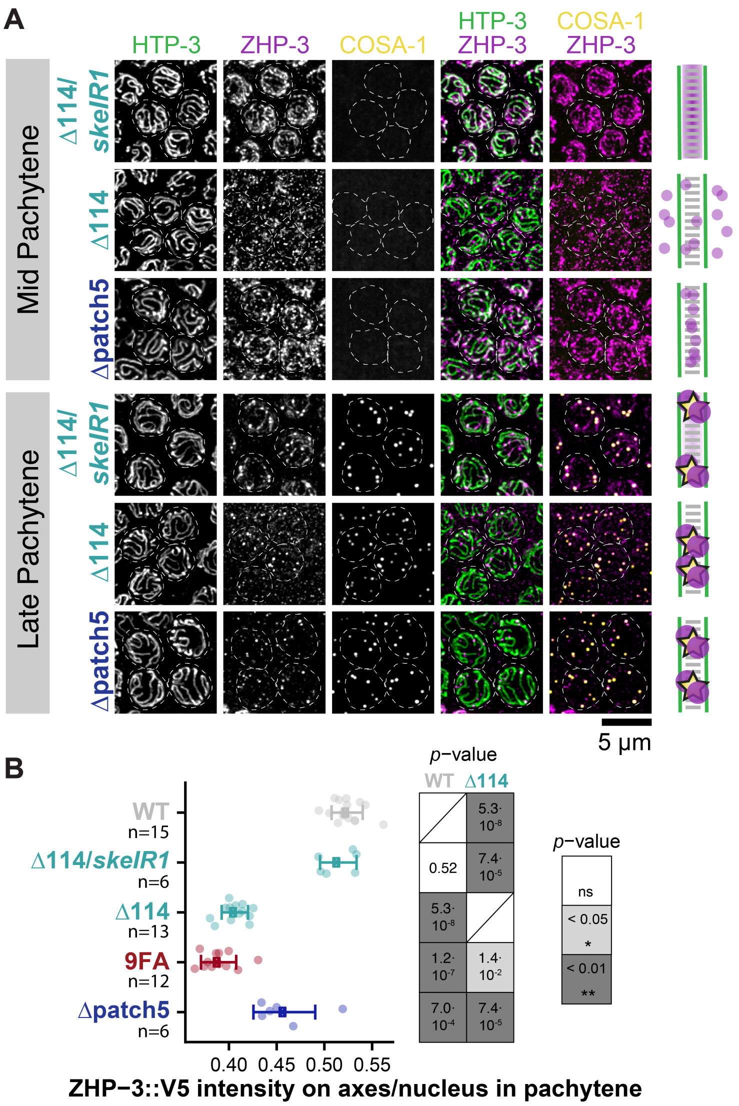

Fig. S11: **ZHP-3 is mislocalised in *syp-4*$^{\Delta 114}$, *syp-4*^{9FA} and *syp-4*$^{\Delta patch5}$ animals.** (A) Maximum intensity projections of mid and late pachytene nuclei stained for the HA-tagged SC SYP-4 (magenta), V5-tagged ZHP-3 (green), and Halo-tagged COSA-1 (yellow) show that ZHP-3 fails to co-localise with the SC in *syp-4*$^{\Delta 114}$ but not *syp-4*$^{\Delta 114}$/*skeIR1* animals, and is decreased along the SC in *syp-4*$^{\Delta patch5}$ animals. Merged images are shown on the right, and cartoons summarise the findings. Nuclei are encircled with a dashed line. (B) The quantification of ZHP-3::V5 loading on the axis relative to the total levels in each nucleus shows that ZHP-3::V5 loads normally in *syp-4*$^{\Delta 114}$/*skeIR1* animals but not in *syp-4*$^{\Delta 114}$, *syp-4*^{9FA}, or *syp-4*$^{\Delta patch5}$ animals. Error bars show mean ± standard deviation. The number of gonads analysed for each condition is given by n.

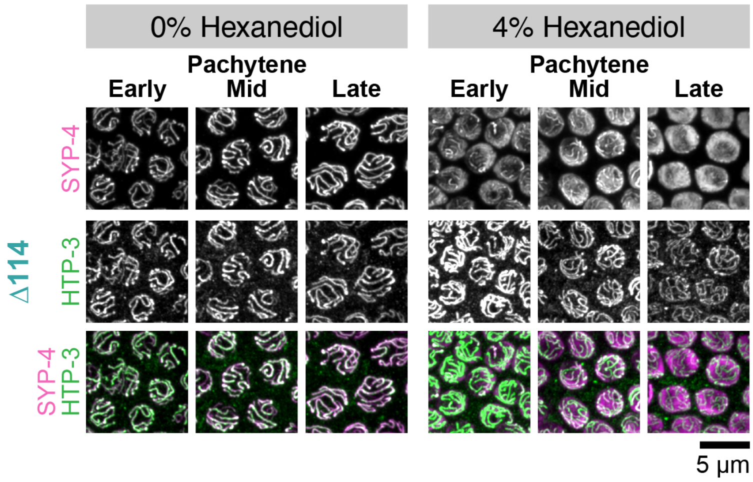

Fig. S12: **The biophysical properties of the SC are altered in *syp-4*$^{\Delta 114}$ animals.** Maximum intensity projections of early, mid and late pachytene nuclei from extruded gonads treated with either 0% (w/v) or 4% (w/v) 1,6-hexanediol and stained for the HA-tagged SC protein SYP-4$^{\Delta 114}$ (magenta, top) and the axis protein HTP-3 (green, middle). The merged image is shown at the bottom.

Table S1. List of *C. elegans* strains used in this study.

Strain Code	Genotype	Source	Referred to as
N2	wild-type	CGC	N2
CB4856	wild-type	CGC	-
SMN440	*syp-4(ie29[syp-4::ha])* I	This study	WT (in Fig. 5B and S3D)
SMN251	*syp-4(ie29[syp-4::ha])* I; *cosa-1(ske25[halo::cosa-1])* III	This study	WT (all remaining Figures)
SMN378	*syp-4(ie29[syp-4::ha])/skeIR1(ske61[let-383 + eft-3p::gfp::NLS::tbb-2 3'UTR]*, CB4856>N2*])* I; *cosa-1(ske25[halo::cosa-1])* III	This study	WT/*skeIR1*
SMN262	*syp-4(ske35-1[syp-4(NP)::ha])* I; *cosa-1(ske25[halo::cosa-1])* III	This study	WT (in Fig. S2)
SMN263	*syp-4(ske35-1[syp-4(NP)::ha])* I/*hT2 [bli-4(e937) let-?(q782) qIs48]* (I;III); *cosa-1(ske25[halo::cosa-1])* III	This study	WT/*hT2*
SMN426	*syp-4(ie29[syp-4::ha]) zhp-3(ske71-1[zhp-3::v5]* I; *cosa-1(ske25[halo::cosa-1])* III	This study	WT (in Fig. 5A)
SMN360-361	*syp-4(ie29[syp-4::ha])* I; *cosa-1(ske25[halo::cosa-1])* III; *rad-51b (ske48[v5::rad-51b])* IV	This study	WT (in Fig. S8)
SMN242	*syp-4(ske19-2[syp-4(Δ114)::ha])* I/*hT2 [bli-4(e937) let-?(q782) qIs48]* (I;III); *cosa-1(ske25[halo::cosa-1])* III	This study	Δ114 (homozygous) or Δ114/*hT2* (heterozygous) (in Fig. S2)
SMN380	*syp-4(ske19-4[syp-4(Δ114)::ha])/skeIR1(ske61[let-383 + eft-3p::gfp::NLS::tbb-2 3'UTR*, CB4856>N2]) I; *cosa-1(ske25[halo::cosa-1])* III	This study	Δ114 (homozygous) or Δ114/*skeIR1* (heterozygous) (all remaining Figures)
SMN438-439	*syp-4(ske19-4[syp-4(Δ114)::ha])/skeIR1(ske61[let-383 + eft-3p::gfp::NLS::tbb-2 3'UTR*, CB4856>N2]) *zhp-3(ske71-2/-3[zhp-3::v5])* I; *cosa-1(ske25[halo::cosa-1])* III	This study	Δ114 (homozygous) (in Fig. S11)
SMN415-416	*syp-4(ske19-4[syp-4(Δ114)::ha])/skeIR1(ske61[let-383 + eft-3p::gfp::NLS::tbb-2 3'UTR*, CB4856>N2]) I; *cosa-1(ske25[halo::cosa-1])* III; *rad-51b (ske48[v5::rad-51b])* IV	This study	Δ114 (homozygous) (in Fig. S8)
SMN421	*syp-4(ske39-1[syp-4(9FA)::ha])/skeIR1(ske61[let-383 + eft-3p::gfp::NLS::tbb-2 3'UTR*, CB4856>N2]) I; *cosa-1(ske25[halo::cosa-1])* III	This study	9FA (homozygous) or 9FA/*skeIR1* (heterozygous) (all remaining Figures)
SMN428-430	*syp-4(ske39-1[syp-4(9FA)::ha])/skeIR1(ske61[let-383 + eft-3p::gfp::NLS::tbb-2 3'UTR]*, CB4856>N2]) *zhp-3(ske71-4/-5/-6[zhp-3::v5])* I; *cosa-1(ske25[halo::cosa-1])* III	This study	9FA (homozygous) (in Fig. 5A)
SMN422	*syp-4(ske39-1[syp-4(9FA)::ha])/skeIR1(ske61[let-383 + eft-3p::gfp::NLS::tbb-2 3'UTR]*, CB4856>N2]) I; *cosa-1(ske25[halo::cosa-1])* III; *rad-51b (ske48[v5::rad-51b])* IV	This study	9FA (homozygous) (in Fig. S8)
SMN464-465	CB4856; *syp-4(ske39-2[syp-4(9FA)::ha])/ske61[let-383 + eft-3p::gfp::NLS::tbb-2 3'UTR]* I	This study	-
SMN292-293	*syp-4(ske36-1/-2[syp-4(10D)::ha])* I; *cosa-1(ske25[halo::cosa-1])* III	This study	10SD
SMN294-298	*syp-4(ske30-1/-2/-3/-4[syp-4(10A)::ha])* I; *cosa-1(ske25[halo::cosa-1])* III	This study	10SA
SMN344	*syp-4(ske37[syp-4(5D)::ha])* I; *cosa-1(ske25[halo::cosa-1])* III	This study	5SD
SMN335	*syp-4(ske38[syp-4(5A)::ha])* I; *cosa-1(ske25[halo::cosa-1])* III	This study	5SA
SMN337-339	*syp-4(ske40-1/-2/-3[syp-4(Δ485-504aa)::ha])* I; *cosa-1(ske25[halo::cosa-1])* III	This study	Δpatch1
SMN340-341	*syp-4(ske41-1/-2[syp-4(Δ514-521aa)::ha])* I; *cosa-1(ske25[halo::cosa-1])* III	This study	Δpatch2
SMN346	*syp-4(ske42-2[syp-4(Δ527-543aa)::ha])* I; *cosa-1(ske25[halo::cosa-1])* III	This study	Δpatch3
SMN342-343	*syp-4(ske43-1/-2[syp-4(Δ559-564aa)::ha])* I; *cosa-1(ske25[halo::cosa-1])* III	This study	Δpatch4
SMN461-463	*syp-4(ske44[syp-4(Δ573-605aa)::ha])* I; *cosa-1(ske25[halo::cosa-1])* III	This study	Δpatch5

Table S2. List of CRISPR RNA sequences used to generate new alleles.

Allele	Target Gene/Allele	crRNA1 Name	crRNA1 Sequence (5'→3')	crRNA2 Name	crRNA2 Sequence (5'→3')
ske35[syp-4(NP)::ha]	*ie29[syp-4::ha]*	crAN7	ggagcaggctgcactctccc	-	-
ske19[syp-4(Δ114)::ha]	*ie29[syp-4::ha]*	crAN10	agagaagttattgcttttgt	crSKie29-61	cataatctgggacatcgtag
ske30[syp-4(10A)::ha]	*ske35[syp-4(NP)::ha]*	crAN13	ttcctgctcttggctcaggg	crSKie29-61	cataatctgggacatcgtag
ske36[syp-4(10D)::ha]	*ske35[syp-4(NP)::ha]*	crAN13	ttcctgctcttggctcaggg	crSKie29-61	cataatctgggacatcgtag
ske37[syp-4(5D)::ha]	*ske35[syp-4(NP)::ha]*	crAN13	ttcctgctcttggctcaggg	crSKie29-61	cataatctgggacatcgtag
ske38[syp-4(5A)::ha]	*ske35[syp-4(NP)::ha]*	crAN13	ttcctgctcttggctcaggg	crSKie29-61	cataatctgggacatcgtag
ske39[syp-4(9FA)::ha]	*ske35[syp-4(NP)::ha]*	crAN13	ttcctgctcttggctcaggg	crSKie29-61	cataatctgggacatcgtag
ske40[syp-4(Δ485-504aa)::ha]	*ske35[syp-4(NP)::ha]*	crAN13	ttcctgctcttggctcaggg	crSKie29-61	cataatctgggacatcgtag
ske41[syp-4(Δ514-521aa)::ha]	*ske35[syp-4(NP)::ha]*	crAN13	ttcctgctcttggctcaggg	crSKie29-61	cataatctgggacatcgtag
ske42[syp-4(Δ527-543aa)::ha]	*ske35[syp-4(NP)::ha]*	crAN13	ttcctgctcttggctcaggg	crSKie29-61	cataatctgggacatcgtag
ske43[syp-4(Δ559-564aa)::ha]	*ske35[syp-4(NP)::ha]*	crAN13	ttcctgctcttggctcaggg	crSKie29-61	cataatctgggacatcgtag
ske44[syp-4(Δ573-605aa)::ha]	*ske35[syp-4(NP)::ha]*	crAN13	ttcctgctcttggctcaggg	crSKie29-61	cataatctgggacatcgtag
ske25[halo::cosa-1]	*cosa-1*	crSK34	aagtgtcaatgtcaagttct	-	-
ske61[let-383 + eft-3p::gfp::NLS::tbb-2 3'UTR]	*let-383*	crAN14	gacgtctcccgcaacatttg	crAN15	gagttagcagctctccagtt
ske71[zhp-3::v5]	*zhp-3*	crAN17	gagattaaaacattaatcgg	-	-
ske48[v5::rad-51b]	*ske1(ha::rad-51b)*	crSKie29-61	cataatctgggacatcgtag	-	-

Table S3. List of template sequences used to generate new alleles.

Allele	Gene Target	Repair Template Sequence (5'→3')
ske35[syp-4(NP)::ha]	*ie29[syp-4::ha]*	caaggacattctagccacggagcaggccgccctgagccaagagcaggaaccagagatcgttgagaagcag
ske19[syp-4(Δ114)::ha]	*ie29[syp-4::ha]*	gatgtagaagacgaagaagaagtgatgagcgaaaacggtagcaacaaatatccctacgatgtcccagatt<br>atgcttagaaaattatcatg
ske30[syp-4(10A)::ha]	*ske35[syp-4(NP)::ha]*	cggtgagcaagaagaaattgagcaaccatctgtattcaaggacattctagccacggagcaggctgcgcta<br>gcccaagaacaagaaccagagatcgttgagaagcaggcagacaatgatgttcagtttgttgatggtaagt<br>gttttacttggcaataaacattgagttattcatttcgcagatcaacaagtgggagcacaactagatgtag<br>aagacgaagaagaagtgatggccgaaaacggatccaacaaaagcaataacttcgcctttaactttttgg<br>gaatagcaagggtacatcagcgggagaaggtggcggtgaaggcagtaagttatgaaacacgtgaaattaa<br>aaaaaactccatttttatagacttcgactttaattttgacggaattggagcaggagatgatggtgccaac<br>aatggtggtgccgccggagatctggatttccttaactatgacggagaggatgaaggaaagggtgccggca<br>acactcaatccgatccgttcggatttgcagccaatggtaacgcagctggtggaggtggagatggagcctt<br>caactttaactttgacggtgacggtgaaggcggagcaactgccggagcgggcggaaacagcacctcgttc<br>tttaacttttatccatatgacgtgcccgattacgcttagaaaattatcatgtattattcagctcttgtat<br>catttgtatcgtttaatgtcagaaattttagattttattggaccaagaaattgcc
ske36[syp-4(10D)::ha]	*ske35[syp-4(NP)::ha]*	cggtgagcaagaagaaattgagcaaccatctgtattcaaggacattctagccacggagcaggctgctcta<br>gaccaagaacaagaaccagagatcgttgagaagcaggcagacaatgatgttcagtttgttgatggtaagt<br>gttttacttggcaataaacattgagttattcatttcgcagatcaacaagtgggagcacaactagatgtag<br>aagacgaagaagaagtgatggacgaaaacggatccaacaaaagcaataacttcgactttaactttttgg<br>gaatagcaagggtacatcagcgggagaaggtggcggtgaaggcagtaagttatgaaacacgtgaaattaa<br>aaaaaactccatttttatagacttcgactttaattttgacggaattggagcaggagatgatggtgacaac<br>aatggtggtgacgacggagatctggatttccttaactatgacggagaggatgaaggaaagggtgacggaa<br>acactcaatccgatccgttcggatttgcagacaatggtaacgcagctggtggaggtggagatggagactt<br>caactttaactttgacggtgacggtgaaggcggagcaactgacggagcgggcggaaacagcacctcgttc<br>tttaacttttatccatatgacgtgcccgattacgcttagaaaattatcatgtattattcagctcttgtat<br>catttgtatcgtttaatgtcagaaattttagattttattggaccaagaaattgcc
ske37[syp-4(5D)::ha]	*ske35[syp-4(NP)::ha]*	cggtgagcaagaagaaattgagcaaccatctgtattcaaggacattctagccacggagcaggctgctcta<br>agccaagaacaagaaccagagatcgttgagaagcaggcagacaatgatgttcagtttgttgatggtaagt<br>gttttacttggcaataaacattgagttattcatttcgcagatcaacaagtgggagcacaactagatgtag<br>aagacgaagaagaagtgatgagcgaaaacggatccaacaaagacaataacttcgactttaactttttgg<br>gaatagcaagggtacatcagcgggagaaggtggcggtgaaggcagtaagttatgaaacacgtgaaattaa<br>aaaaaactccatttttatagacttcgactttaattttgacggaattggagcaggagatgatggttcgaac<br>aatggtggttctactggagatctggatttccttaactatgacggagaggatgaaggaaagggctcaggaa<br>acactcaagacgatccgttcggatttgcatcaaatggtaacgcagctggtggaggtggagatggagactt<br>caactttaactttgacggtgacggtgaaggcggagcaacttcaggcgctggcggaaacgacacctcgttc<br>tttaacttttatccctacgatgtcccagattatgcttagaaaattatcatgtattattcagctcttgtat<br>catttgtatcgtttaatgtcagaaattttagattttattggaccaagaaattgcc
ske38[syp-4(5A)::ha]	*ske35[syp-4(NP)::ha]*	cggtgagcaagaagaaattgagcaaccatctgtattcaaggacattctagccacggagcaggctgctcta<br>agccaagaacaagaaccagagatcgttgagaagcaggcagacaatgatgttcagtttgttgatggtaagt<br>gttttacttggcaataaacattgagttattcatttcgcagatcaacaagtgggagcacaactagatgtag<br>aagacgaagaagaagtgatgagcgaaaacggatccaacaaagccaataacttcgcctttaactttttgg<br>gaatagcaagggtacatcagcgggagaaggtggcggtgaaggcagtaagttatgaaacacgtgaaattaa<br>aaaaaactccatttttatagacttcgactttaattttgacggaattggagcaggagatgatggttcgaac<br>aatggtggttctactggagatctggatttccttaactatgacggagaggatgaaggaaagggctcaggaa<br>acactcaagccgatccgttcggatttgcatcaaatggtaacgcagctggtggaggtggagatggagcctt<br>caactttaactttgacggtgacggtgaaggcggagcaacttcaggcgctggcggaaacgccacctcgttc<br>tttaacttttatccctacgatgtcccagattatgcttagaaaattatcatgtattattcagctcttgtat<br>catttgtatcgtttaatgtcagaaattttagattttattggaccaagaaattgcc
ske39[syp-4(9FA)::ha]	*ske35[syp-4(NP)::ha]*	cggtgagcaagaagaaattgagcaaccatctgtattcaaggacattctagccacggagcaggctgctcta<br>agccaagaacaagaaccagagatcgttgagaagcaggcagacaatgatgttcagtttgttgatggtaagt<br>gttttacttggcaataaacattgagttattcatttcgcagatcaacaagtgggagcacaactagatgtag<br>aagacgaagaagaagtgatgagcgaaaacggatccaacaaaagcaataacttctctgccaacgccgccgg<br>gaatagcaagggtacatcagcgggagaaggtggcggtgaaggcagtaagttatgaaacacgtgaaattaa<br>aaaaaactccatttttatagacttcgacgccaatgccgacggaattggagcaggagatgatggttcgaac<br>aatggtggttctactggagatctggatgcccttaactatgacggagaggatgaaggaaagggctcaggaa<br>acactcaatccgatccgttcggatttgcatcaaatggtaacgcagctggtggaggtggagatggatcgtt<br>caactttaactttgacggtgacggtgaaggcggagcaacttcaggcgctggcggaaacagcacctcggcc<br>gccaacgcctatccctacgatgtcccagattatgcttagaaaattatcatgtattattcagctcttgtat<br>catttgtatcgtttaatgtcagaaattttagattttattggaccaagaaattgcc

Continued on next page

Table S3 – *Continued from previous page*

Allele	Target Gene/Allele	Repair Template Sequence (5'→3')
ske40[syp-4(Δ485-504aa)::ha]	*ske35[syp-4(NP)::ha]*	cggtgagcaagaagaaattgagcaaccatctgtattcaaggacattctagccacggagcaggctgctcta<br>agccaagaacaagaaccagagatcgttgagaagcaggcagacaatgatgttcagtttgttgatggtaagt<br>gttttacttggcaataaacattgagttattcatttcgcagatcaacaagtgggagcacaactagatgtag<br>aagacgaagaagaagtgatgggtacatcagcgggagaaggtggcggtgaaggcagtaagttatgaaacac<br>gtgaaattaaaaaaaactccatttttatagacttcgactttaattttgacggaattggagcaggagatga<br>tggttcgaacaatggtggttctactggagatctggatttccttaactatgacggagaggatgaaggaaag<br>ggctcaggaaacactcaatccgatccgttcggatttgcatcaaatggtaacgcagctggtggaggtggag<br>atggatcgttcaactttaactttgacggtgacggtgaaggcggagcaacttcaggcgctggcggaaacag<br>cacctcgttctttaacttttatccctacgatgtcccagattatgcttagaaaattatcatgtattattca<br>gctcttgtatcatttgtatcgtttaatgtcagaaattttagattttattggaccaagaaattgcc
ske41[syp-4(Δ514-521aa)::ha]	*ske35[syp-4(NP)::ha]*	cggtgagcaagaagaaattgagcaaccatctgtattcaaggacattctagccacggagcaggctgctcta<br>agccaagaacaagaaccagagatcgttgagaagcaggcagacaatgatgttcagtttgttgatggtaagt<br>gttttacttggcaataaacattgagttattcatttcgcagatcaacaagtgggagcacaactagatgtag<br>aagacgaagaagaagtgatgagcgaaaacggatccaacaaaagcaataacttctcttttaactttttgg<br>gaatagcaagggtacatcagcgggagaaggtggcggtgtaagttatgaaacacgtgaaattaaaaaaaac<br>tccatttttataggacggaattggagcaggagatgatggttcgaacaatggtggttctactggagatctg<br>gatttccttaactatgacggagaggatgaaggaaagggctcaggaaacactcaatccgatccgttcggat<br>ttgcatcaaatggtaacgcagctggtggaggtggagatggatcgttcaactttaactttgacggtgacgg<br>tgaaggcggagcaacttcaggcgctggcggaaacagcacctcgttctttaacttttatccctacgatgtc<br>ccagattatgcttagaaaattatcatgtattattcagctcttgtatcatttgtatcgtttaatgtcagaa<br>attttagattttattggaccaagaaattgcc
ske42[syp-4(Δ527-543aa)::ha]	*ske35[syp-4(NP)::ha]*	cggtgagcaagaagaaattgagcaaccatctgtattcaaggacattctagccacggagcaggctgctcta<br>agccaagaacaagaaccagagatcgttgagaagcaggcagacaatgatgttcagtttgttgatggtaagt<br>gttttacttggcaataaacattgagttattcatttcgcagatcaacaagtgggagcacaactagatgtag<br>aagacgaagaagaagtgatgagcgaaaacggatccaacaaaagcaataacttctcttttaactttttgg<br>gaatagcaagggtacatcagcgggagaaggtggcggtgaaggcagtaagttatgaaacacgtgaaattaa<br>aaaaaactccatttttatagacttcgactttaattttgacggaattggagcaaactatgacggagaggat<br>gaaggaaagggctcaggaaacactcaatccgatccgttcggatttgcatcaaatggtaacgcagctggtg<br>gaggtggagatggatcgttcaactttaactttgacggtgacggtgaaggcggagcaacttcaggcgctgg<br>cggaaacagcacctcgttctttaacttttatccctacgatgtcccagattatgcttagaaaattatcatg<br>tattattcagctcttgtatcatttgtatcgtttaatgtcagaaattttagattttattggaccaagaaat tgcc
ske43[syp-4(Δ559-564aa)::ha]	*ske35[syp-4(NP)::ha]*	cggtgagcaagaagaaattgagcaaccatctgtattcaaggacattctagccacggagcaggctgctcta<br>agccaagaacaagaaccagagatcgttgagaagcaggcagacaatgatgttcagtttgttgatggtaagt<br>gttttacttggcaataaacattgagttattcatttcgcagatcaacaagtgggagcacaactagatgtag<br>aagacgaagaagaagtgatgagcgaaaacggatccaacaaaagcaataacttctcttttaactttttgg<br>gaatagcaagggtacatcagcgggagaaggtggcggtgaaggcagtaagttatgaaacacgtgaaattaa<br>aaaaaactccatttttatagacttcgactttaattttgacggaattggagcaggagatgatggttcgaac<br>aatggtggttctactggagatctggatttccttaactatgacggagaggatgaaggaaagggctcaggaa<br>acactcaagcatcaaatggtaacgcagctggtggaggtggagatggatcgttcaactttaactttgacgg<br>tgacggtgaaggcggagcaacttcaggcgctggcggaaacagcacctcgttctttaacttttatccctac<br>gatgtcccagattatgcttagaaaattatcatgtattattcagctcttgtatcatttgtatcgtttaatg<br>tcagaaattttagattttattggaccaagaaattgcc
ske44[syp-4(Δ573-605aa)::ha]	*ske35[syp-4(NP)::ha]*	cggtgagcaagaagaaattgagcaaccatctgtattcaaggacattctagccacggagcaggctgctcta<br>agccaagaacaagaaccagagatcgttgagaagcaggcagacaatgatgttcagtttgttgatggtaagt<br>gttttacttggcaataaacattgagttattcatttcgcagatcaacaagtgggagcacaactagatgtag<br>aagacgaagaagaagtgatgagcgaaaacggatccaacaaaagcaataacttctcttttaactttttgg<br>gaatagcaagggtacatcagcgggagaaggtggcggtgaaggcagtaagttatgaaacacgtgaaattaa<br>aaaaaactccatttttatagacttcgactttaattttgacggaattggagcaggagatgatggttcgaac<br>aatggtggttctactggagatctggatttccttaactatgacggagaggatgaaggaaagggctcaggaa<br>acactcaatccgatccgttcggatttgcatcaaatggtaacgcagctggttatccctacgatgtcccaga<br>ttatgcttagaaaattatcatgtattattcagctcttgtatcatttgtatcgtttaatgtcagaaatttt<br>agattttattggaccaagaaattgcc
ske25[halo::cosa-1]	*cosa-1*	(btn)cagtgaaaaatcgtgaaaactgaactgaagtgtcaatggccgagatcggaaccggattcccattc<br>gacccacactacgtcgaggtccttggagagcgcatgcactacgtcgacgtcggaccacgcgacggaaccc<br>cagtccttttccttcacggaaacccaacctcctcctacgtctggcgcaacatcatcccacacgtcgcccc<br>aacccaccgctgcatcgccccagaccttatcggaatgggaaagtccgacaagccagaccttggatacttc<br>ttcgacgaccacgtccgtttcatggacgccttcatcgaggcccttggacttgaggaggtcgtccttgtca<br>tccacgactggggatccgcccttggattccactgggccaagcgcaacccagagcgcgtcaagggaatcgc<br>cttcatggagttcatccgcccaatcccaacctgggacgagtggccagagttcgcccgcgagaccttccaa<br>gccttccgcaccaccgacgtcggacgtaagcttatcatcgaccaaaacgtcttcatcgagggaacccttc<br>caatgggagtcgtccgtccacttaccgaggtcgagatggaccactaccgcgagccattccttaacccagt<br>cgaccgcgagccactttggcgcttcccaaacgagcttccaatcgccggagagccagccaacatcgtcgcc<br>cttgtcgaggagtacatggactggcttcaccaatccccagtcccaaagcttcttttctggggaaccccag<br>gagtccttatcccaccagccgaggccgcccgtcttgccaagtcccttccaaactgcaaggccgtcgacat<br>cggaccaggacttaaccttcttcaagaggacaacccagaccttatcggatccgagatcgcccgttggctt<br>tccacccttgagatctccggaggaggaggaagttcttcacggtgagttgtcgtttcaaaataaaatgcga acactgc

Continued on next page

Table S3 – *Continued from previous page*

Allele	Target Gene/Allele	Repair Template Sequence (5'→3')
ske61[let-383 + eft-3p::gfp::NLS::tbb-2 3'UTR]	*let-383*	ctttttccgccagcacggacgtctcccgcaacatgcacctttggtcttttattgtcaacttccattggt<br>tcttccattgtttctgttaaattaatgaatttttcataaaataaagacattatacaatataaaaatgaag<br>aatttattgaaaataaactgccagagagaaaaagtatgcaacactcccgccgagagtgtttgaaatggtg<br>tacggtacattttcgtgctaggagttagatgtgcaggcagcaacgagagggggagagatttttttgggcc<br>ttgtgaaattaacgtgagttttctggtcatctgactaatcatgttggtttttttgttggtttattttgttt<br>ttatctttgtttttatccagattaggaaatttaaattttatgaatttataatgaggtcaaacattcagtc<br>ccagcgtttttcctgttctcactgtttagtcgaatttttattttaggctttcaacaaatgttctaactgt<br>cttatttgtgacctcactttttatattttttttaatttttaaaaatattagaagtttctaggataattttt<br>tcgacttttattctctctaccgtccgcactcttcttacttttaaattaaattgttttttttttcagttggg<br>aaacactttgctcactccgtagcagccatggcaagtttgtacaaaaaagcaggctcgccaaagaagaagc<br>gtaaggtgtccaaggtaagttttcttatgggaaagaaggaaaaaaccgagattttacttgaaaaattgaa<br>tttttcgcgggattttcaccaaaaattgttgaatattcattatttcacgctgtaaaacaaaaaaaaaaaa<br>aatcaaaaactacgttgaaatcgcgtttttaagcgaattttcttcagaattgccagattttaaccccaaa<br>ttttgcagtttttaaataaaatttcacctttcggctcaaattgtagattttcctgaaaatttagtacaa<br>aaaacaatttcctcgtaaatttttcaaatagattttcagggagaggagctcttcaccggagtcgtcccaa<br>tcctcgtcgagctcgacggagacgtcaacggacacaagttctccgtctccggagagggagagggagacgc<br>cacctacggaaagctcaccctcaagttcatctgcaccaccggaaagctcccagtcccatggccaaccctc<br>gtcaccaccttctgctacggagtccaatgcttctcccgttacccagaccacatgaagcgtcacgacttct<br>tcaagtccgccatgccagagggatacgtccaagagcgtaccatcttcttcgtaagttatgtctacctgcc<br>tgcctaccgcctaaattttttgtgaagtttcttcaaaaaatccagaaaaaaaaacaattttcatacgatt<br>ttttcccttaaaattgtgaattttcatgctttttagccccaaaagtcattatttgagaaaaaatttcata<br>caaaaaagttttgagaaatacacaatttttttaaatgtaattttcaaattttcaattttcaactagaaaat<br>tcacaaaacttgtaaattttggaccaaaacatttatacaattacttttttttgaatctaataactacaata<br>actacaaatttaacattttcagaaggacgacggaaactacaagacccgtgccgaggtcaagttcgaggga<br>gacaccctcgtcaaccgtatcgagctcaagggaatcgacttcaaggaggacggaaacatcctcggacaca<br>agctcgagtacaactacaactcccacaacgtctacatcatggccgacaagcaaaagaacggaatcaaggt<br>caacttcaaggtaagttcttttttttgaaaagtgcagtttgtagtctaattttcatttattttctttttaa<br>aaaacgcatcaatttttaaatattttgggacaaaatccgaaaactgtactaatttgtagtttgtaaaatt<br>aaaaaaaaaacgcaaaaaaatgttttcaaaaatgcttagaataaaaataaaatttctaaaaaattgaaca<br>aaataaacatttaaaaattcaaaaagtttgaaaaaatgcaaagtttttttaatgcaaaaaatatttaatgt<br>ttaaacaaatttaaaaaatgttgtaagaatatgtttagagacagtttttaaatgttaaattgcaaaaaaa<br>aaaaagcaaaaaaaaaatttgaaaaatgtaacagaaaattttaattaaaaaagtcgtgtttttttcaaacc<br>tgcaattctttgtaattttatttataaagagaaacaaatcaaattgtatattacgaaaaacgatactaaa<br>attcgaaaatttgcggtttttttgcgtaaaaaatacggttccgtaattttcagatccgtcacaacatcgag<br>gacggatccgtccaactcgccgaccactaccaacaaaacaccccaatcggagacggaccagtcctcctcc<br>cagacaaccactacctctccacccaatccgccctctccaaggtaagttcatagattttgaaaaaaagttt<br>aagaactgaaaaatggaataaaaatatttaagagcatttttaaatgtaaaattaacaaaaaaagcgccta<br>agaatgtttccaaaacagtaaaaaaaaggtttaaaaaaatgcaaaaaaaaatttaagatgcttttaatta<br>cacaaaaaatgaaagtaaaaaaaattaaaatattgaaaaatgtatatttgtttgaaaaggtacatttttc<br>aatgcaaaaaatgtttctaaaaattaaaaaaatatgtttaaattgtgtaaagtgtaaattaaaaaataaa<br>attatgaaaaagtattgtttcatactttttctaaattttggttgaaaaatttaaaattccagctttttat<br>tcacattatgtgttctaaaaatctttccccaattttcgggaccgttttccagtatatttccatttttcaa<br>aaagatttttaactgaaattcatgttttcaatgctaaaaatcaataaaaaaagaatttttcaggacccaa<br>acgagaagcgtgaccacatggtcctcctcgagttcgtcaccgccgccggaatcacccacggaatggacga<br>gctctacaagtcccgtcgtcgtaaggccaacccaaccaagctctccgagaacgccaagaagctcgccaag<br>gaggtcgagaacaacccagctttcttgtacaaagtgggataaatgcaaaatcctttcaagcattcccttc<br>ttctctatcactcttctttctttttgtcaaaaaattctctcgctaatttatttgctttttttaatgttatt<br>attttatgactttttatagtcactgaaaagtttgcatctgagtgaagtgaatgctatcaaaatgtgattc<br>tgtctgatgtactttcacaatctctcttcaattccattttgaagtgctttaaacccgaaaggttgagaaa<br>aatgcgagcgctcaaatatttgtattgtgttcgttgagtgacccaacaaaaagaggaaactttattgtgc<br>cgccaagaaaaaagtctcagttaggttatttttatatgtatatatatatattta
ske71[zhp-3::v5]	*zhp-3*	gaaaccgatcaatggtcggagcttcattggacccgccgatggaggaaagccaattccaaacccacttctt<br>ggactcgactccacctaatgttttaatctcgtttttttctgaattcgttc
ske48[v5::rad-51b]	*ske1[ha::rad-51b]*	cttaaaaataattaattaaaattatctcaggagtcaaaaatgggaaagccaatcccaaacccacttctt<br>ggactcgactccacctcagcacaagcaagtcgtcaaaagaaatcggatcaa

Table S4. List of primer sequences and restriction enzymes used for genotyping the new alleles. For the detection of silent mutations, restriction sites were added (+) or removed (-) in the new allele.

**Allele**	**Forward Primer**	**Sequence (5'→3')**	**Reverse Primer**	**Sequence (5'→3')**	**Restriction sites**
ske19[syp-4(Δ114)::ha]	AN45	gaatcaatcctagatgagcccgaag	OR236	gaggcaatttcttggtccaataa	-
ske30[syp-4(10A)::ha]	AN45	gaatcaatcctagatgagcccgaag	OR236	gaggcaatttcttggtccaataa	HaeIII (-); BamHI (+); XmnI (-)
ske36[syp-4(10D)::ha]	AN45	gaatcaatcctagatgagcccgaag	OR236	gaggcaatttcttggtccaataa	HaeIII (-); BamHI (+); XmnI (-)
ske37[syp-4(5D)::ha]	AN45	gaatcaatcctagatgagcccgaag	OR236	gaggcaatttcttggtccaataa	HaeIII (-); BamHI (+); XmnI (-)
ske38[syp-4(5A)::ha]	AN45	gaatcaatcctagatgagcccgaag	OR236	gaggcaatttcttggtccaataa	HaeIII (-); BamHI (+); XmnI (-)
ske39[syp-4(9FA)::ha]	AN45	gaatcaatcctagatgagcccgaag	OR236	gaggcaatttcttggtccaataa	HaeIII (-); BamHI (+); XmnI (-)
ske40[syp-4(Δ485-504aa)::ha]	AN45	gaatcaatcctagatgagcccgaag	OR236	gaggcaatttcttggtccaataa	HaeIII (-); XmnI (-)
ske41[syp-4(Δ514-521aa)::ha]	AN45	gaatcaatcctagatgagcccgaag	OR236	gaggcaatttcttggtccaataa	HaeIII (-); BamHI (+); XmnI (-)
ske42[syp-4(Δ527-543aa)::ha]	AN45	gaatcaatcctagatgagcccgaag	OR236	gaggcaatttcttggtccaataa	HaeIII (-); BamHI (+); XmnI (-)
ske43[syp-4(Δ559-564aa)::ha]	AN45	gaatcaatcctagatgagcccgaag	OR236	gaggcaatttcttggtccaataa	HaeIII (-); BamHI (+); XmnI (-)
ske44[syp-4(Δ573-605aa)::ha]	AN45	gaatcaatcctagatgagcccgaag	OR236	gaggcaatttcttggtccaataa	HaeIII (-); BamHI (+); XmnI (-)
ske25[halo::cosa-1]	SK449	gtattggtctcgcaccgaac	SK450	ctgctgatacggcaggtgta	-
ske61[let-383 + eft-3p::gfp::NLS::tbb-2 3'UTR]	AN80	ctctgtccaactgtacccattc	AN82	ctgcacatctaactcctagcac	-
ske61[let-383 + eft-3p::gfp::NLS::tbb-2 3'UTR]	AN85	gagaagcgtgaccacatgg	AN86	cagatggaaatgagagaaacaggc	-
ske71[zhp-3::v5]	IC101	ctcactctctcattccggg	IC102	cagatgtgaactaggtagagaaaaag	-
ske[v5::rad-51b]	IC99	gctccactgttaaaaatgccg	IC100	atatcgccagaactgattcctg	-

Table S5. Embryonic viability and male progeny for each of the strains used in this study. The mean embryonic lethality and male progeny, and the corresponding standard deviation (SD), are given for each strain. *P*-values were calculated using the Mann-Whitney test and adjusted using the Benjamini-Hochberg method for comparing the embryonic lethality and male progeny of each strain to N2. Strains containing epitope-tagged wild-type genes are marked by a †. Statistical significance: * p-value<0.05, ** p-value<0.01, *** p-value<0.001.

**Strain Code**	**Genotype**	**#Parents**	**#Eggs**	**#Adults**	**#Males**	**Embryonic Lethality**		**Male Progeny**	
						Mean±SD(%)	Adjusted *p*-value	Mean±SD(%)	Adjusted *p*-value
N2	wild-type	43	10229	10837	8	-6.7 ± 8.2	-	0.1 ± 0.2	-
CB4856	wild-type	17	3050	3121	2	-1.2 ± 22.0	2.58E-01	0.1 ± 0.2	3.23E-01
SMN440 †	*syp-4(ie29[syp-4::ha])* I	7	1478	1610	1	-9.2 ± 3.9	2.17E-01	0.1 ± 0.1	8.71E-01
SMN251 †	*syp-4(ie29[syp-4::ha])* I; *cosa-1(ske25[halo::cosa-1])* III	24	5540	5646	4	-2.4 ± 6.1	5.78E-02	0.1 ± 0.2	9.45E-01
SMN378	*syp-4(ie29[syp-4::ha])/skeIR1(ske61[let-383 + eft-3p::gfp::NLS::tbb-2 3'UTR*, CB4856>N2]) I; *cosa-1(ske25[halo::cosa-1])* III	4	969	734	0	24.8 ± 5.7	2.06E-03 **	0 ± 0	4.00E-01
SMN262 †	*syp-4(ske35-1[syp-4(NP)::ha])* I; *cosa-1(ske25[halo::cosa-1])* III	6	1277	1272	3	0.6 ± 8.5	8.50E-02	0.2 ± 0.4	3.23E-01
SMN263	*syp-4(ske35-1[syp-4(NP)::ha])* I/*hT2 [bli-4(e937) let-?(q782) qIs48]* (I;III); *cosa-1(ske25[halo::cosa-1])* III	5	756	182	0	76.1 ± 1.6	7.31E-04 ***	0 ± 0	3.55E-01
SMN426 †	*syp-4(ie29[syp-4::ha]) zhp-3(ske71-1[zhp-3::v5]* I; *cosa-1(ske25[halo::cosa-1])* III	5	1061	1206	7	-13.8 ± 10.4	1.71E-01	0.5 ± 0.5	1.54E-02 *
SMN360-361†	*syp-4(ie29[syp-4::ha])* I; *cosa-1(ske25[halo::cosa-1])* III; *rad-51b (ske48[v5::rad-51b])* IV	15	3575	3828	26	-7.4 ± 6.4	5.70E-0.1	0.7 ± 0.7	3.46E-05***
SMN242	*syp-4(ske19-2[syp-4(Δ114)::ha])* I; *cosa-1(ske25[halo::cosa-1])* III	9	990	86	32	88.5 ± 12.7	1.04E-05 ***	32.0 ± 25.4	5.27E-05 ***
SMN242	*syp-4(ske19-2[syp-4(Δ114)::ha])* I/*hT2* (I;III); *cosa-1(ske25[halo::cosa-1])* III	14	2314	364	18	85.6 ± 5.1	2.03E-07 ***	4.6 ± 5.3	1.77E-04 ***
SMN380	*syp-4(ske19-4[syp-4(Δ114)::ha])* I; *cosa-1(ske25[halo::cosa-1])* III	14	2402	218	85	90.5 ± 3.0	2.03E-07 ***	39.7 ± 10.5	2.50E-09 ***
SMN380	*syp-4(ske19-4[syp-4(Δ114)::ha])/skeIR1(ske61[let-383 + eft-3p::gfp::NLS::tbb-2 3'UTR*, CB4856>N2]) I; *cosa-1(ske25[halo::cosa-1])* III	9	2180	1622	6	25.2 ± 8.1	1.04E-05 ***	0.4 ± 0.6	4.88E-02 *
SMN438-439	*syp-4(ske19-4[syp-4(Δ114)::ha]) zhp-3(ske71-2/-3[zhp-3::v5])* I; *cosa-1(ske25[halo::cosa-1])* III	4	394	53	24	87.8 ± 3.0	2.06E-03 **	42.3 ± 16.6	5.60E-05 ***
SMN415-416	*syp-4(ske19-4[syp-4(Δ114)::ha])* I; *cosa-1(ske25[halo::cosa-1])* III; *rad-51b (ske48[v5::rad-51b])* IV	5	842	106	39	87.6 ± 2.5	6.85E-04***	39.2 ± 9.8	1.18E-05***
SMN421	*syp-4(ske39-1[syp-4(9FA)::ha])* I; *cosa-1(ske25[halo::cosa-1])* III	4	670	62	16	91.8 ± 6.4	2.06E-03 **	20.1 ± 13.7	4.48E-03 **
SMN428-430	*syp-4(ske39-1[syp-4(9FA)::ha]) zhp-3(ske71-4/-5/-6[zhp-3::v5])* I; *cosa-1(ske25[halo::cosa-1])* III	16	3422	502	188	85.6 ± 3.3	1.13E-07 ***	37.4 ± 9.1	1.10E-09 ***
SMN422	*syp-4(ske39-1[syp-4(9FA)::ha])* I; *cosa-1(ske25[halo::cosa-1])* III; *rad-51b (ske48[v5::rad-51b])* IV	5	1106	131	51	88.2 ± 1.9	6.85E-04***	38.3 ± 11.7	1.18E-05***
SMN464-465	CB4856; *syp-4(ske39-2[syp-4(9FA)::ha]* I	7	1536	218	46	85.8 ± 2.7	7.31E-05 ***	21.4 ± 4.8	9.19E-07 ***
SMN292-293	*syp-4(ske36-1/-2[syp-4(10D)::ha])* I; *cosa-1(ske25[halo::cosa-1])* III	10	2140	1968	51	8.5 ± 4.7	2.14E-05 ***	2.8 ± 1.3	3.62E-08 ***
SMN294-298	*syp-4(ske30-1/-2/-3/-4[syp-4(10A)::ha])* I; *cosa-1(ske25[halo::cosa-1])* III	20	3605	3417	42	5.9 ± 16.0	7.38E-06 ***	1.1 ± 0.8	5.28E-09 ***
SMN344	*syp-4(ske37[syp-4(5D)::ha])* I; *cosa-1(ske25[halo::cosa-1])* III	7	1600	1575	10	0.5 ± 7.9	8.30E-02	0.6 ± 0.6	9.73E-03 **
SMN335	*syp-4(ske38[syp-4(5A)::ha])* I; *cosa-1(ske25[halo::cosa-1])* III	8	1917	1819	10	3.8 ± 10.2	6.12E-03 **	0.5 ± 0.3	1.24E-05 ***
SMN337-339	*syp-4(ske40-1/-2/-3[syp-4(Δ485-504aa)::ha])* I; *cosa-1(ske25[halo::cosa-1])* III	11	2621	2658	8	-1.8 ± 5.2	6.40E-02	0.3 ± 0.3	8.35E-03 **
SMN340-341	*syp-4(ske41-1/-2[syp-4(Δ514-521aa)::ha])* I; *cosa-1(ske25[halo::cosa-1])* III	6	1187	1188	5	1.2 ± 6.7	4.12E-02 *	0.4 ± 0.4	8.22E-03 **
SMN346	*syp-4(ske42-2[syp-4(Δ527-543aa)::ha])* I; *cosa-1(ske25[halo::cosa-1])* III	8	1566	1573	9	1.5 ± 8.7	2.86E-02 *	0.6 ± 1.1	1.62E-01
SMN342-343	*syp-4(ske43-1/-2[syp-4(Δ559-564aa)::ha])* I; *cosa-1(ske25[halo::cosa-1])* III	11	1938	1953	8	1.3 ± 11.1	2.86E-02 *	0.4 ± 0.4	8.35E-03 **
SMN461-463	*syp-4(ske44[syp-4(Δ573-605aa)::ha])* I; *cosa-1(ske25[halo::cosa-1])* III	10	2250	1389	71	38.0 ± 6.9	6.48E-06 ***	5.3 ± 2.1	3.62E-08 ***

Table S6. List of *Caenorhabditis* species used for SYP-4 multiple sequence alignment.

**Species**	**Protein Assembly**	**Ortholog**
C. elegans	WS285	H27M09.3
C. becei	WS285	CSP29.g2226.t1
C. brenneri	WS285	CBN08735
C. briggsae	WS285	CBG04214
C. inopinata	WS285	Sp34_10146410.t1
C. inopinata	WS285	Sp34_10147300.t1
C. latens	WS285	FL83_17238
C. nigoni	WS285	Cni-syp-4
C. panamensis	WS285	CSP28.g9372.t1
C. remanei	WS285	CRE01249
C. sinica	WS285	Csp5_scaffold_00925.g16811.t1
C. sulstoni	WS285	CSP32.g1474.t1
C. sulstoni	WS285	CSP32.g1476.t1
C. tribulationis	WS285	CSP40.g15409.t1
C. tropicalis	WS285	Csp11.Scaffold627.g6892.t1
C. uteleia	WS285	CSP31.g22718.t1
C. waitukubuli	WS285	CSP39.g827.t1
C. zanzibari	WS285	CSP26.g17156.t1

Table S7. Total number of peptides containing each serine and threonine residue from position 440 and number of peptides containing a phosphorylated form of serine/threonine at the respective position. A total of 3 experiments were performed. * marks residues mutated in 10SD/10SA animals and † marks residues mutated in 5SD/5SA animals.

Position	Amino Acid	#Peptides covering amino acid residue	#Peptides with phosphorylated amino acid residue
441	T	14	0
447*	S	12	4
485*	S	10	5
489	S	10	0
492†	S	15	0
496*†	S	5	1
503	S	11	0
506	T	6	0
507	S	6	0
531*	S	0	0
536*	S	0	0
537*	T	0	0
554*	S	17	5
557	T	17	0
559†	S	17	0
566*	S	0	0
578*†	S	0	0
592	T	5	0
593*	S	5	0
599†	S	5	0
600	T	5	0
601	S	5	0
